# Adipose-derived mesenchymal stem cells for the treatment of Amyotrophic Lateral Sclerosis. A phase I/II safety and efficacy clinical trial

**DOI:** 10.3389/fneur.2025.1655124

**Published:** 2025-10-08

**Authors:** Eduardo Agüera-Morales, Victoria Eugenia Fernández-Sánchez, Guillermo Navarro-Mascarell, Juan Antonio Cabezas-Rodríguez, María Ángeles Peña-Toledo, Virginia Reyes-Garrido, María José Postigo-Pozo, Giorgio Patrignani-Ochoa, María Ángeles Geniz-Clavijo, Celedonio Márquez-Infante, Luis Tallon-Aguilar, José Tinoco-González, Javier Padillo-Ruiz, Amador Valladares-Sánchez, Candela Caballero-Eraso, Cecilia López-Ramírez, Rosario Mata Alcázar-Caballero, Laura Leyva-Fernández, Antonio Rodríguez-Acosta, Rafael Maldonado-Sánchez, María Luisa García-Martín, MªLuisa Somoza-Ramírez, Blanca Quijano-Ruiz, María del Mar Macías-Sánchez, Gloria Carmona-Sánchez, Olga Fernández-López, Óscar Fernández-Fernández

**Affiliations:** 1 Neurology Unit, Reina Sofía University Hospital, Córdoba, Spain; 2 Clinical Neurophysiology Service, Málaga Regional University Hospital, Málaga, Spain; 3 Neurology Unit, Virgen Macarena University Hospital, Seville, Spain; 4 Neuromuscular Unit, Virgen del Rocío University Hospital, Seville, Spain; 5 Neurology Service, Málaga Regional University Hospital, Málaga, Spain; 6 General and Gastroenterology Surgery Service, Virgen del Rocío University Hospital, Seville, Spain; 7 Pneumology and Thorax Surgery Unit, Virgen del Rocío University Hospital, Seville, Spain; 8 Andalusian Network for the Design and Translation of Advanced Therapies, Andalusian Progress and Health Public Foundation, Seville, Spain; 9 Department of Pharmacology and Pediatrics, Medical School, Málaga University, Málaga, Spain; 10 Cell Production Unit, Málaga Regional University Hospital, Málaga, Spain; 11 Nuclear Magnetic Resonance Service, Biomedical Research Institute of Málaga and Nanomedicine Platform, Málaga, Spain

**Keywords:** amyotrophic lateral sclerosis, cell therapy, mesenchymal stem cells, adipose-derived mesenchymal stem cells, neurodegeneration, clinical trial

## Abstract

**Introduction:**

Amyotrophic lateral sclerosis (ALS) is a progressive and fatal neurodegenerative disease with few treatments available. Mesenchymal stem cells have arisen as a potential treatment option for ALS due to their immune system modulation and their neuroprotective effects. This clinical trial aimed to evaluate the safety, efficacy and feasibility of three intravenous doses of autologous adipose-derived mesenchymal stem cells (AdMSC) in ALS patients.

**Methods:**

A multicentre, randomized, parallel group, placebo-controlled, double-blinded clinical trial (EudraCT: 2011-006254-85) was conducted in 40 patients with ALS in treatment with riluzole. Patients were randomized 1:1:1:1 into the following treatment groups: 1 × 10^6^ cells/kg, 2 × 10^6^ cells/kg, 4 × 10^6^ cells/kg and placebo. After a 6 month follow-up, patients in the placebo group were randomized 1:1:1 to receive one of the three doses of AdMSC and they were followed up for another 6 months. Lastly, all patients were followed-up in a 36-month open-label extension. Safety was mainly assessed through the evaluation of adverse events and their relationship with the medicinal product. Several variables were measured to assess efficacy, such as ALS Functional Rating Scale, Ashworth spasticity scale, neurophysiological and neuropsychological parameters and overall survival. The feasibility of the procedure was assessed through the evaluation of the extraction and infusion of AdMSC.

**Results:**

Safety of AdMSC was observed through all follow-up periods, with similar percentages of adverse events between groups and no significant differences between groups in the rate of adverse events related to treatment. The administration procedure was feasible for all patients. Across all analyzed measures, we observed the expected progressive decline characteristic of ALS, with no statistically significant between-group differences in the rate of change.

**Discussion:**

The results obtained in this study are consistent with the ones obtained in other clinical trials using similar doses of MSC, where safety was demonstrated and efficacy results were inconclusive, due to not reaching statistical significance. Larger studies with an increased sample size, different doses and route of administration or combination of routes, repeated dosing or larger duration and comprehensive assessment of immunological effect would be needed to analyze the efficacy of AdMSC in the treatment of ALS.

**Clinical trial registration:**

https://www.clinicaltrialsregister.eu/ctr-search/search?query=2011-006254-85.

## 1 Introduction

Amyotrophic Lateral Sclerosis (ALS) represents one of the most devastating neurodegenerative diseases, characterized by the progressive degeneration of motor neurons in the brain and spinal cord. This loss of neurons leads to muscle weakness, atrophy and, eventually, paralysis, severely affecting patients’ quality of life. Usually, death occurs three to 5 years after the diagnosis of the disease, although certain varieties show prolonged survival (1). In 90% of the cases, ALS is sporadic and with no known cause; the 10% of remaining cases are familial and linked to the transmission of mutations in genes that have a wide range of functions, including the functionality of non-motor cells. The hereditary form of ALS is most commonly due to a G4C2 hexanucleotide repeat expansion in C9orf72. Other established high-penetrance genes include SOD1, TARDBP, and FUS, with additional contributions from OPTN, VCP, SQSTM1, TBK1, UBQLN2, VAPB, KIF5A, NEK1, MATR3, CHCHD10, TUBA4A, ANXA11, CCNF, and C21orf2. These discoveries implicate pathways spanning RNA metabolism/protein homeostasis, axonal transport/cytoskeleton, and innate immunity (1–6).

ALS presents a homogeneous incidence rate of about 1.7 patients per 100,000 population per year and an overall worldwide prevalence of 4.42 patients per 1,00,000 population (7, 8). The number of cases in 2018 in the United States were estimated to be 29,824, making it a rare disease by the Food Drug Administration (less than 200,000 people in the United States) (9, 10), and it is also considered a rare disease in the European Union (less than 5 per 10,000 in the Community) (11).

Although motor neuron degeneration is ultimately responsible for ALS, it is widely recognized as a complex and multifactorial condition. In recent years the important role and involvement of the adaptive and innate immune system in the pathogenesis of the disease has become evident, in both mouse models and ALS patients (6).

The participation of the humoral immune system has been reflected in several studies. On one hand, increased levels of circulating immunocomplexes and IgG have been found in the serum of ALS patients (12, 13). Some patients present antibodies against voltage-dependent calcium channels (14) and anti-GM1 or anti-GD1 ganglioside antibodies in higher titers than healthy controls, although their significance is unknown (15). It has also been found that ALS patients with longer survival were reported to have IgM antibodies against the mutant SOD1 protein form (16). In patients with sporadic ALS it has also been shown that their immunoglobulins are capable of producing calcium-dependent apoptosis via an oxidative damage mechanism (17). It has also been observed that immunoglobulins from ALS patients passively transferred to an ALS murine model increased glutamate levels in the cerebrospinal fluid (CSF) of rats (18).

Furthermore, in relation with the innate immune system response, microglial activation and proliferation occur early during the development of ALS, particularly in areas of significant motor neuron loss (19, 20). However, the mechanisms by which microglial cells are involved in motor neuron death in ALS are not fully understood. It is known that microglial activation increases throughout the course of the disease and is associated with an altered production of toxic and neurotrophic factors. In addition, it seems that the adaptive immune system may also influence the course of the disease (21–23).

Despite advances in the understanding of its pathogenesis, the treatment of ALS remains a considerable challenge and there are few treatments available to slow the progression of the disease. Riluzole, the only drug approved for this indication in Europe, extends survival in ALS patients only by 2–3 months and increases the chance of an additional year of survival by ~9%, so that the disease normally leads to death 2 to 4 years after its onset (24, 25). The urgency of developing more effective and personalized therapies is therefore of paramount importance.

The multiple mechanisms involved in the immune response to ALS and their potential as drug targets are currently being explored. In relation to this approach, mesenchymal stem cells (MSC) have arisen as a potential treatment option. MSC are non-hematopoietic stromal cells, which can be obtained from various sources, i.e., bone marrow (BM-MSC) or adipose tissue (AdMSC—adipose derived mesenchymal stem cells). Aside from their classical role of supporting hematopoiesis and producing cells of the mesodermal lineage, additional properties including immunomodulatory and neurotrophic effects, have been described (26–30). AdMSC exhibit similar properties to BM-MSC and are isolated from the stromal vascular fraction of adipose tissue, presenting the advantage that the samples are extractable with a minimally invasive lipectomy procedure and they can provide a higher number of MSC than bone marrow samples. Furthermore, AdMSC have shown a higher proliferation capacity and can be maintained *in vitro* for extended periods with a stable population. Moreover, adipose tissue provides a higher number of MSC than bone marrow in samples of equal mass (31, 32).

Adipose derived mesenchymal stem cells directly administrated into the CSF have shown a protective effect on motor neurons and a reduction in glial activation, both in vitro (ALS astrocytes-motor neuron co-cultures) and *in vivo* models (33). Specifically, a delay of motor decline and prolonged survival in the SOD1-G93A murine model of ALS has been observed after the systemic administration of adipose derived mesenchymal stem cells (34). In another study with superoxide-dismutase 1 (SOD1)-mutant transgenic mice, the animal model of familial ALS, a combination of intrathecal and intramuscular MSC administered to these animals exhibited an increase in motor neuron survival, maintained neuromuscular junctions in quadriceps femoris and showed a substantial reduction involved in necroptosis, apoptosis and autophagy (35). Up-regulation in levels of glial-derived neurotrophic factor (GDNF) and basic fibroblast growth factor (bFGF) has also been observed in familial ALS mice models (34, 36). Recent studies suggest that intravenous (IV) administration of MSCs acts primarily through a transient, paracrine ‘hit-and-run’ mechanism. After a brief pulmonary first-pass, IV-infused MSCs are licensed by inflammatory cytokines and release extracellular vesicles, cytokines and mitochondria that re-programme peripheral immune cells (37–39).

Several clinical trials have shown that the injection of MSC is safe and well tolerated in ALS patients through different routes of administration, such as direct injection in CSF (40–42) and spinal cord (43), intramuscular injection (44) or intravenous injection (45). Studies involving patients with multiple sclerosis have also demonstrated safety and signs of efficacy, suggesting neuroprotection (46–48). Also, a clinical trial using repeated intrathecal injections of MSC in ALS patients reported additional transient clinical benefit (45). Nonetheless, the results of these studies are difficult to interpret due to the heterogeneity of their designs and the low number of patients recruited. In addition, the optimal type of stem cells to be used (bone marrow, fat, dental pulp, etc.) and the ideal route of administration are not clearly established. Furthermore, no double-blind trial had evaluated three escalating IV doses of adipose derived MSC with long-term follow-up, which is a gap highlighted in consensus reviews (49). The characteristics of AdMSCs made them the chosen investigational product for the present clinical trial, mainly due to its minimally invasive extraction process, the high number of cells obtained with an extraction of limited tissue. The investigational product was administered intravenously to promote their action through a transient, paracrine “hit-and-run” mechanism that reprograms peripheral immunity after a brief pulmonary first pass (37–39). The present study, a safety-anchored phase I/II design with long-term follow-up, using adipose derived mesenchymal stem cells, aims to provide more information regarding these aspects, confirm the safety of the treatment and investigate its efficacy in ALS patients.

## 2 Methods

### 2.1 Study design

The present study was a phase I/II multicentre, randomized, parallel group, placebo-controlled, double blinded clinical trial in patients with moderate to severe Amyotrophic Lateral Sclerosis (ALS). It was conducted in 4 sites in Spain, from July 2014 to March 2022: the Málaga Regional University Hospital, the Virgen Macarena University Hospital (Seville), the Virgen del Rocío University Hospital (Seville) and the Reina Sofía University Hospital (Córdoba). The Biomedical Research Institute of Málaga and Nanomedicine Platform was responsible for the metabolomic analysis of the samples.

The study was conducted in accordance with European legislation (Directive 2001/20/EC) and Spanish legislation (Royal Decree 223/2004 and Royal Decree 1090/2015) in force at that time, following all the ethical principles of the Declaration of Helsinki and the Good Clinical Practices defined by the International Council for Harmonisation of Technical Requirements for Pharmaceuticals for Human Use (ICH).

Spanish regulatory authorities and the Ethics Committee of Sevilla approved the clinical trial and all amendments made during the course of the study.

#### 2.1.1 Study treatment

Four treatment groups were included in the study: three experimental groups, consisting of a single dose of adipose-derived mesenchymal stem cells (AdMSC) (1 × 10^6^ cells/kg, 2 × 10^6^ cells/kg and 4 × 10^6^ cells/kg), and one control arm, consisting of a single dose of placebo. Treatment was assigned to each participant by a simple randomization procedure in a 1:1:1:1 ratio, with no stratification.

#### 2.1.2 Study stages

**Figure 1:**
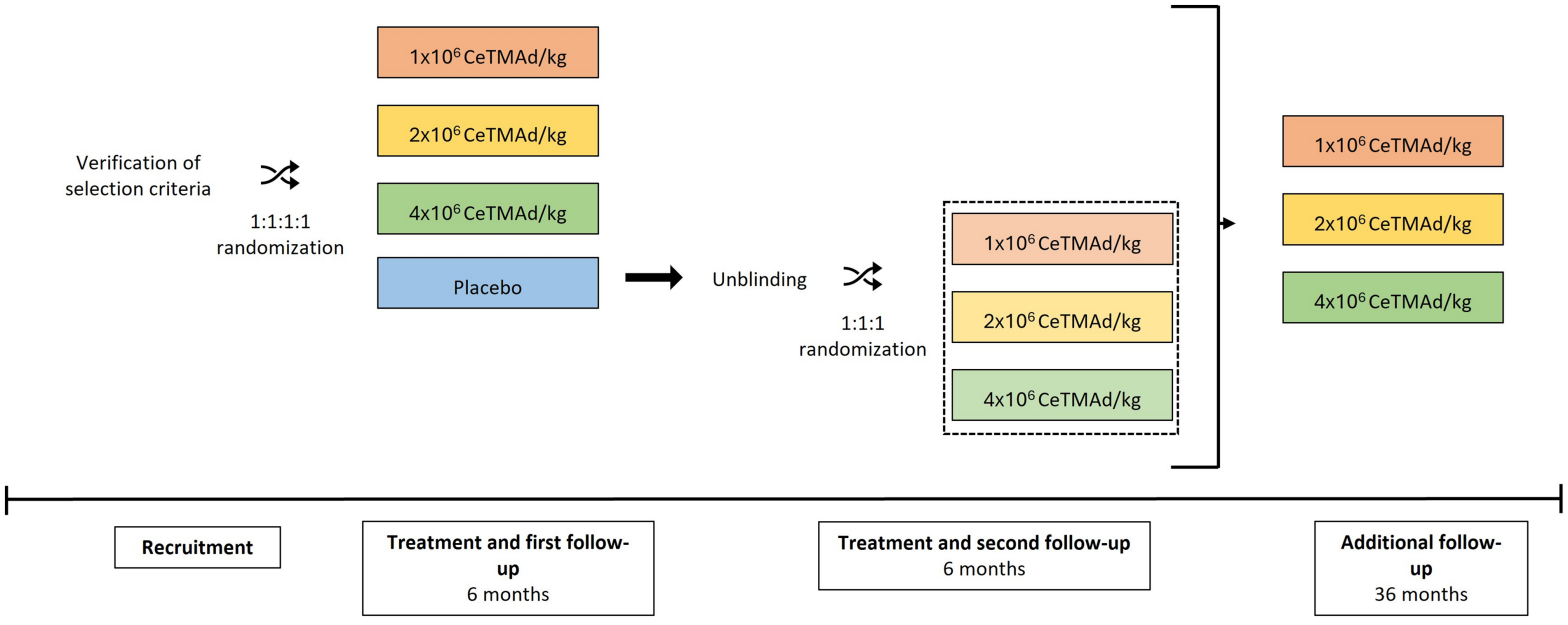
Trial schema and timelines. CONSORT-style flow of screening, 1:1:1:1 randomization to placebo or AdMSC at 1 × 10^6^, 2 × 10^6^, or 4 × 10^6^ cells/kg, blinded 6-month follow-up, re-randomization of the placebo group to active doses, second 6-month follow-up, and a 36-month open-label extension.

The study was divided into four main periods (Figure 1). The first period was the recruitment (90 days), in which participants meeting all the inclusion criteria and none of the exclusion criteria were randomized to one of the four treatment arms. Medical history was recorded and general physical and neurological explorations, as well as a specific ALS evaluation, were performed. Blood was also collected for biochemical, hematological and immunological studies. Lastly, adipose tissue was extracted for the manufacture of AdMSC.

The next period (follow-up) began with the administration of the treatment assigned to each patient and was divided into two follow-up periods. The first follow-up lasted 6 months, in which patients could have received either placebo or AdMSC. At the time that each patient completed this first follow-up, unblinding was performed and those patients randomized to the control group were randomized again to one of the three experimental arms. A second follow-up of 6 months began for those patients exclusively.

Lastly, once each of the patients included in the trial completed their 6-month follow-up after having received the experimental treatment, they continued in an open-label extension study for 36 months to evaluate the long-term safety of AdMSC.

Hence, for patients initially assigned to the experimental treatment, the study lasted 45 months (90 days of pre-inclusion period, 6 months of initial follow-up and 36 months of additional follow-up during the extension study). For patients initially assigned to the placebo group, the study lasted 51 months (90 days of pre-inclusion period, 6 months of initial follow-up, 6 months of follow-up after administration of the experimental treatment and 36 months of additional follow-up during the extension study).

### 2.2 Participants

40 adults (≥18 years old) with a diagnosis of probable or definite Amyotrophic Lateral Sclerosis, according to the El Escorial criteria of the World Federation of Neurology (50), with an evolution of the disease from the onset of symptoms of more than 6 and less than 36 months, and in treatment with riluzole for at least 1 month prior to inclusion, were selected. Patients with previous stem cell therapy were excluded. Detailed eligibility criteria are provided in Table 1. Written informed consent was obtained from all participants.

**Table 1:** Inclusion, exclusion and withdrawal criteria.

Inclusion criteria	Female and male adults over 18 years old. Good understanding of the protocol and ability to give informed consent. Diagnosis of sporadic ALS, with a diagnosis of certainty, i.e., definite or probable, according to the El Escorial criteria of the World Federation of Neurology. Forced vital capacity of at least 50% of that which would correspond to their sex, height and age. More than 6 and less than 36 months of evolution of the disease (from the onset of symptoms). Possibility of obtaining at least 50 grams of adipose tissue. Treatment with riluzole for at least one month prior to inclusion.
Exclusion criteria	Any concomitant disease that at the investigator’s criteria could affect the measurements of the clinical variables of the trial (hepatic, renal or cardiac insufficiency, diabetes mellitus, etc.). Previous stem cell therapy. Participation in another clinical trial during the 3 months prior to entry into this clinical trial. Any lymphoproliferative disease. Tracheostomy and/or gastrostomy. Hemophilia, hemorrhagic diathesis or current anticoagulant therapy (provided that medical-surgical criteria advise against its temporary withdrawal prior to procedures for which withdrawal of anticoagulant therapy is necessary). Known hypersensitivity to fetal bovine serum or gentamicin. HIV infection (positive HIV antibody) or any severe immunocompromised state. Positive HCV serology (positive anti-HCV). Active HBV infection (positive HBsAg). Serum creatinine levels >3.0 in patients not subjected to hemodialysis. Alcohol or drug addiction. Pregnancy, planning to become pregnant or patients of childbearing age who are not under birth control methods. Lactation. Any other condition, for which, in the judgement of the principal investigator, the subject is considered unsuitable for the study.
Withdrawal criteria	Presence of serious adverse event from inclusion of the patient in the clinical trial (signature of the informed consent) to the date of the administration of the investigational medicinal product or placebo, provided that, in the opinion of the investigator and/or the sponsor, the safety of the patient is at risk or could interfere with the interpretation of the study results. If the required final cell concentration of the investigational product is not achieved, the sponsor, in consultation with the investigator, will decide whether to proceed with administration or reschedule manufacturing. In cases where it has not been possible to obtain at least 50 g of adipose tissue, the sponsor will decide whether the subject remains in the study. In the event of an incident occurring during the manufacturing process of the investigational product that prevents its infusion, it will be possible to consider, to criterion of the responsible doctor, the execution of a new extraction of adipose tissue to the patient. In this case it would be mandatory for the patient to give informed consent for this second extraction. In this case, the patient will keep their allocation number and randomization group. When the patient does not cooperate or does not comply with the requirements of the study. Clinical conditions of the patient at any time during the development of the trial that prevent its continuity. Necessity, at medical discretion, of the use of clinical alternatives excluded from the protocol of this clinical trial. Abnormal laboratory values or any test required in the protocol, whenever, in the opinion of the investigator and/or sponsor, they jeopardize the patient’s safety or may interfere with the interpretation of the results of the study. Violation of the protocol, whenever, in the judgement of the investigator and/or sponsor, the patient’s safety is at risk or may interfere with the interpretation of the results of the study. Withdrawal of consent by the patient. Loss of patient follow-up. Grade IV toxicity on the WHO scale of adverse reactions. When the responsible investigator considers that the patient’s health is compromised due to adverse reactions, concomitant diseases or any other circumstances that may arise during the study.

### 2.3 Trial procedures

The investigational medicinal product (IMP) was a suspension of autologous mesenchymal stem cells obtained from adipose tissue (AdMSCs), manufactured at the Málaga Regional University Hospital (Spain) from adipose tissue received within 12 h of its extraction. Briefly, after mechanical disruption, the tissue was digested for 60–90 min with 0.3 IU/g collagenase NB6 (Nordmark) in sterile tubes at 37 °C with agitation. A 1:1 ratio of expansion medium [glucose reduced Dulbecco’s modified Eagle’s medium (Sigma-Aldrich) supplemented with 2% L-alanyl-L-glutamine (Sigma-Aldrich), 10% fetal bovine serum (SAFC Biosciences) and 0.1 mg/mL gentamicin (Normon Laboratories)] was added to inactivate the enzyme activity before centrifugation at 600 *g* for 10 min. The cells were collected, filtered, seeded (at 10–20 × 10^4^ nucleated viable cells/cm^2^) and maintained as previously described (51) using expansion medium.

AdMSCs were passaged at ≥80% confluency and re-seeded at 2.5 -5 × 10^3^ cells/cm^²^ for a maximum of seven passages to obtain the IMP dose of 1 × 10^6^, 2 × 10^6^ AdMSCs or 4 × 10^6^ AdMSC/kg of patient body mass and were then suspended in 100 mL of lactated Ringer solution (Baxter Laboratories) supplemented with 2.5% glucose (Braun Medical) and 1% human serum albumin (Grifols Laboratories), packaged into two 50 mL Luer-Lock cone syringes (Becton Dickinson) and sent to the appropriate center for infusion at 2–20 °C monitored temperature (within 16 h). AdMSCs obtained from the patients randomized to placebo were expanded and cryopreserved for their administration in the second follow-up period.

All manufactured IMPs met the finished product specifications such as sterility, AdMSC characterization (phenotype), viability, endotoxins, cell doublings (passages), karyotype and mycoplasma detection.

The three doses of AdMSC (1 × 10^6^ ± 10%, 2 × 10^6^ ± 10% and 4 × 10^6^ ± 10% cells/kg) were administered intravenously, in order to evaluate whether there is a direct relationship between dose and therapeutic efficacy, knowing that these doses have been safe when administered intravenously in other studies. Most of the accumulated clinical data come from treatments in hematological diseases, where doses vary from 1 to 5 million cells per kg (28). Several consensus groups recommend doses in the range of 1–3 million cells per kg, suggesting that higher doses would probably be more effective (52, 53).

All patients in the trial received riluzole as baseline therapy at the approved dosing regimen (50 mg tablets taken orally twice daily). Because no other active comparator was available, placebo was chosen as the control treatment, which consisted in 100 mL of Ringer’s lactate solution supplemented with 2.5% glucose and 1% albumin. Both placebo and stem cells were administered in a single dose to each patient by intravenous infusion over 120 min at a 50 mL/h flow rate. At baseline and follow-up visits, information of potential trial outcomes, adverse events and concomitant therapies were collected.

### 2.4 Primary and secondary endpoints

#### 2.4.1 Primary endpoints

The main objective of the study was to determine the safety of the intravenous (IV) administration of the three doses of AdMSC. Primary endpoints related to this objective were: adverse events and their causal relationship with the medicinal product and the administration procedure, appearance of new neurological deficits not attributed to the natural history of ALS, and lastly, complications at the infusion site.

#### 2.4.2 Secondary endpoints

##### 2.4.2.1 Efficacy

The secondary objectives were the assessment of the efficacy and the immunomodulatory effects of the administration of AdMSC compared to placebo. Efficacy was measured using several variables so that the clinical improvement and progression of the disease were properly comprehended. Regarding the evaluation of efficacy, the following variables were assessed.

First, changes in the speed of disease progression were measured through changes in the ALS Functional Rating Scale – Revised (ALSFRS-R) after 1, 3 and 6 months during the first and second follow-up, and every 3 months during the additional follow-up. This scale examines the patient disability by area and consists of 12 items grouped into 4 functionality domains: bulbar (items of speech, salivation and swallowing), fine motor (handwriting, cutting food, dressing and hygiene), gross motor (turning in bed, walking and climbing stairs) and respiratory (dyspnea, orthopnea and respiratory insufficiency). After calculation of the domains, the ALSFRS-R scale generates stages defined as follows: stage 1—functional impairment, but with independence in all domains; stage 2—dependence in one domain; stage 3—dependence in two domains; stage 4—dependence in three domains; stage 5—dependence in all four domains (54). Changes in the total scoring, changes in the domains and clinical improvement were analyzed. As for clinical improvement, an increase in total scores by 20% from baseline levels was considered clinically significant (55).

On the other hand, modifications in muscle strength grade were measured through manual muscle testing (MMT) using the Medical Research Council (MRC) scale. This method allows the analysis of muscle strength in 34 areas or muscle groups. The patient is observed, and values are assigned from 0 to 5: 0—no visible contraction; 1—visible muscle contraction, but no movement of the limb; 2—active movement, but not against gravity; 3—active movement against gravity; 4—active movement against gravity and resistance; 5—active movement with full resistance (56). This evaluation has ordinal scale scores in each area, but the total score (average of the scores of all areas) is quantitative. This score is the one analyzed in this study, evaluating changes each month.

Improvements in forced vital capacity (FVC) were measured through spirometry every 3 months. A decrease of FVC in 10% or more with respect to the previous value was considered a worsening and progression of ALS. A decrease of less than 10%, maintenance or increase of FVC was considered an improvement and, therefore, no progression of ALS.

Furthermore, magnetic resonance imaging (MRI) with a 1.5 Tesla equipment was used to estimate the changes in muscle mass of upper and lower limbs. The volume of muscle mass of the most affected limb segment in each patient was quantified. This was selected according to the results obtained previously through the neurophysiological study performed during the screening. Bony prominences were used as a reference point for the exploration according to the segment under study. The analysis of changes in muscle mass was performed individually per patient, always comparing the same segment and the same limb in each patient, 6 months after baseline. In the patients initially assigned to placebo group, muscle mass was also assessed 6 months after receiving stem cell treatment.

Changes in circumference of upper and lower limbs were assessed in the right and left arm, forearm, thigh and leg 1, 3 and 6 months since baseline (for patients initially randomized to the placebo group also 1, 3 and 6 months after receiving stem cell therapy). For their evaluation, the average circumference of the left and right limbs was calculated. If the patient only had data for one side, the available data was used.

In addition, neurophysiological parameters were measured through the evolution in the Neurophysiological Index, the Motor Unit Number Estimation (MUNE) and the amplitude and excitability threshold of Motor Evoked Potentials (MEPs) at 6 months since baseline. The neurophysiological index was performed with an electromyograph with a 4-channel amplifier and temperature control. The M and F response was measured after 20 supramaximal electrical stimuli at a frequency of 1 Hz, constant current and duration of 0.1 ms. The ulnar border wrist was stimulated and recorded in the little finger abductor muscle. The neurophysiological index is the result of dividing the amplitude of the M wave/distal motor latency by the frequency of the F wave. MUNE is an estimate of functional motor units, which decreases with disease progression, and is therefore considered a marker of ALS progression (57). MUNE was evaluated with a 4-channel amplified electromyograph in the abductor muscle of the little finger, using incremental technique and with temperature control. MEPs were obtained by monopulse magnetic stimulation and recorded with an electromyograph with software for evoked potentials with a 4-channel amplifier. The methodology applied was as described in the guidelines of the International Federation of Clinical Neurophysiology (58). MUNE and MEPs were analyzed by individual patient comparisons of baseline measurements with successive measurements, due to the fact that patients may be at different points in ALS progression. In this pathology, any change in the described measurements is considered to be clinically significant. Unfavorable progression was defined as a decrease in this measurement with respect to the baseline value, whereas favorable progression was defined as an increase with respect to the baseline value.

Continuing with neuropsychological parameters, they were measured through changes in the Wechsler Adult Intelligence Scale (WAIS-III) at 6 months since baseline. This scale consists of 14 subtests, and it allows to evaluate global intelligence in adults (59). Subtests were individually analyzed for this study.

Changes in quality-of-life parameters were assessed through changes in the Sickness Impact Profile (SIP) (60) at 6 months since baseline. This questionnaire contains 12 categories and a total of 136 items. The overall maximum score for this test is 100%, where a zero represents a good health status without physical or behavioral changes due to illness, while the 100 represents a poor health status or a major impact of illness on behavior.

The evolution of the spasticity was estimated through changes in the Ashworth spasticity scale 1, 3 and 6 months since baseline (for patients initially randomized to the placebo group also 1, 3 and 6 months after receiving stem cell therapy). With this scale, the patient is observed and values from 0 to 4 are assigned (normal muscle tone, mild, intense and extreme hypertonia) for right and left elbows, wrists, knees and ankles (61).

The visual analogue scale (VAS) and the McGill Pain Questionnaire were used to estimate the variation of pain. They were measured 1, 3 and 6 months after baseline (for patients initially randomized to the placebo group also 1, 3 and 6 months after receiving stem cell therapy). Signs of efficacy or improvement in the VAS scale are present when there is no change in the scale, or if there is a decrease of one or more points on the scale. The McGill Questionnaire is a self-reporting measure of pain that assesses both quality and intensity of subjective pain. It has three scale consists of three indices: the pain rating index (PRI), based on two types of numerical values that can be assigned to each word descriptor; the number of words chosen; and the present pain intensity (PPI) based on a 1–5 intensity scale (62).

Furthermore, the need to perform a gastrostomy and the time until it was performed were analyzed. Both parameters were analyzed taking into account the initial randomization groups and the dose of stem cell therapy received (including the patients in the placebo group in the stem cell dose group to which they were randomized after unblinding). The need to perform a tracheostomy or for permanent assisted ventilation and the time until they were performed were also analyzed.

Overall survival was analyzed considering the initial randomization group and the dose of stem cell therapy received, during the active treatment period (time was counted from the time when all patients have received their dose of stem cell therapy) and since baseline. Overall survival according to the region of origin of ALS symptoms (bulbar or pyramidal) was also analyzed.

##### 2.4.2.2 Immunomodulatory effects

Regarding the immunomodulatory effects of AdMSC, they were assessed through metabolomic analysis of CSF by ^1^H NMR spectroscopy. Spectra were acquired on a Bruker Avance III 600 MHz spectrometer using a nitrogen-cooled reverse detection cryoprobe (Prodigy TCI). Water-suppressed 1D Nuclear Overhauser Effect Spectroscopy (NOESY) spectra were acquired for CSF. The acquisition was performed under standard operating procedures.

Metabolite quantification was performed with Chenomx (v 8.4, Chenomx Inc., Edmont, Ca) using Electronic Reference To access In-vivo Concentrations (ERETIC) as the concentration reference, implemented as ERETIC 2 in TopSpin 3.5pl7 (Bruker BioSpin, Ettlingen, Germany).

##### 2.4.2.3 Feasibility of the procedure

Lastly, the feasibility of the procedure was assessed through the evaluation of the percentage of patients in whom the complete infusion procedure (either AdMSC or placebo) could be performed. The study of the feasibility of the procedure was not an objective initially contemplated in the design and development of the trial; rather, it was carried out later with the data obtained during the study.

### 2.5 Statistical analysis

Data analysis was conducted using R statistical software (63) with the IDE RStudio (version 2023.06.2). All general inferential procedures used *α* = 0.05 as the assumed level of risk, excepting the case of adjustments for multiple comparisons.

#### 2.5.1 Statistical analysis of efficacy and feasibility

Efficacy and feasibility analyses were carried out on the intention to treat (ITT) population, consisting of all randomized participants (intention-to-treat set).

For the efficacy analysis, a descriptive and a subsequent inferential analysis was carried out. ANOVA mixed model was used for assessing the changes in disease progression velocity ALSFRS-R scale and its domains, muscle strength grade on MMT, Forced Vital Capacity (FVC%), pain in McGill questionnaire and neurophysiological index, spasticity (Ashworth scale), pain measure through VAS scale, Sickness Impact Profile and need for gastrostomy. Survival and time to gastrostomy were analyzed with Cox regression method. Changes in extremities circumference and neuropsychological parameters (WAIS-III test) were analyzed in a descriptive manner only. Changes in muscular mass, MUNE and MEP were analyzed individually in a descriptive manner for each patient.

^1^H NMR spectra of metabolites were obtained on 18 CSF samples from 9 patients who received AdMSC and completed the first follow-up (each patient had two samples: before infusion and after infusion). Given the small number of samples, a profiling approach was adopted for the analysis, individually quantifying all detectable metabolites (~45) prior to statistical analysis. Another 3 samples from patients who received placebo and completed the first follow-up period were eventually excluded to ensure a better quality of the analysis. Statistical analysis was performed using mixed models with restricted maximum likelihood (REML), assuming a normal distribution of the metabolites. Additionally, paired t-tests and the Wilcoxon test were conducted to compare predictions. The fixed effects in the mixed model included treatment, symptom origin, age and sex, while subject was treated as a random effect.

In the feasibility analysis, the percentage of patients in whom the complete infusion procedure could be performed was evaluated. The infusion procedure was considered feasible when the administration of the cell product or placebo was successfully completed from a technical point of view, in at least 80% of the patients.

#### 2.5.2 Statistical analysis of safety

Safety analysis was carried out on the safety population (SP), consisting of all randomized patients who received the cell product and/or placebo. For this study, SP consisted of the same patients as the ITT population. Adverse Events (AEs), Serious Adverse Events (SAEs), Serious Adverse Reactions (SARs) and Suspected Unexpected Serious Adverse Reactions (SUSARs) were collected. They were coded according to the MedDRA classification (versions 25.1 and 26). A descriptive analysis (frequencies and percentages) of AEs, SAEs and SARs was performed. In addition, the presence of AEs and SAEs associated with the extraction of adipose tissue or with the treatment with AdMSC was analyzed. As for the inferential analysis, statistical significance was studied between the randomization group and the intensity and relationship with the treatment of AEs and SAEs, by means of the chi-square test of association between categorical variables.

## 3 Results

### 3.1 Recruitment and baseline characteristics

From July 2014 to July 2018, a total of 48 patients were recruited, across 4 sites in Spain. 40 patients were finally enrolled in the study; the 8 patients not selected were excluded from the study due to not meeting inclusion criteria (*n* = 5), consent withdrawal (*n* = 1), death (*n* = 1) and an incidence during the fabrication of the investigational product that prevented its administration (*n* = 1).

After recruitment and screening, selected patients were assigned by a simple randomization method to one of the four treatment groups. Two patients were initially assigned to the higher dose of AdMSC, but incidences during the manufacturing of the final product prevented reaching the needed dose. Those patients were included in the previous dose (2 × 10^6^ AdMSC/kg). One patient, initially assigned to the higher dose of AdMSC (4 × 10^6^/kg) received a dose of 2.9 × 10^6^ AdMSC/kg given that the assigned dose was not reached during the fabrication of the product.

Apart from two patients in the medium dose group, all the patients completed the first follow-up period. After unblinding, the 10 patients in the placebo group were randomized to receive one of the 3 doses of stem cell treatment, distributed as follows: 1 × 10^6^ AdMSC/kg (*n* = 4), 2 × 10^6^ AdMSC/kg (*n* = 3) and 4 × 10^6^ AdMSC/kg (*n* = 3). All the patients from the lower and higher dose completed the second follow-up period, while none from the medium dose did.

**Figure 2:**
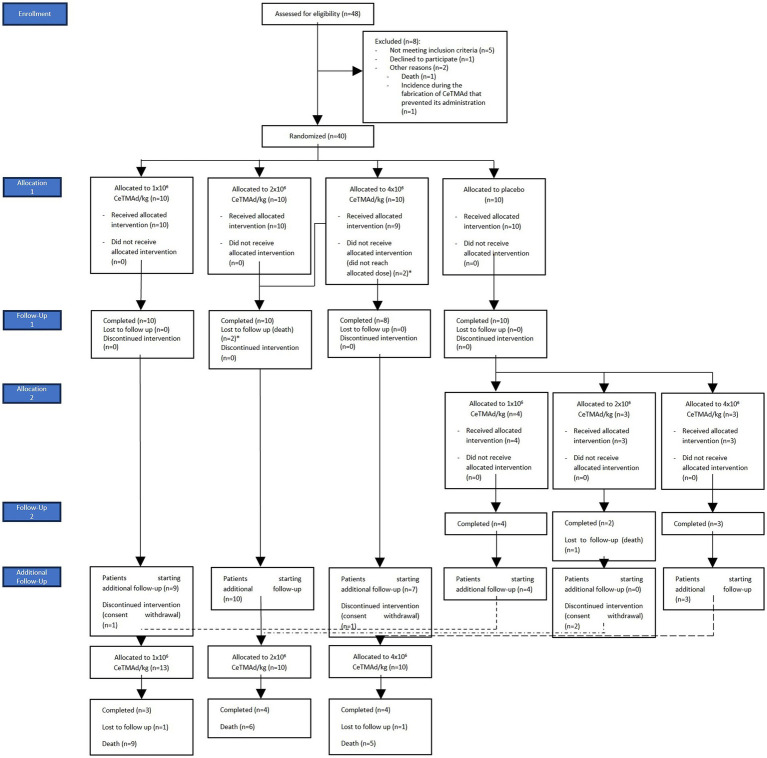
Participant disposition. CONSORT diagram showing 48 assessed, 8 excluded, 40 randomized to placebo or AdMSC (1 × 10^6^, 2 × 10^6^, 4 × 10^6^ cells/kg), follow-up completions, withdrawals, and losses, plus allocation in the additional follow-up.

A total of 33 patients started the additional follow-up period: 13 patients from the 1 × 10^6^ group, 10 from the 2 × 10^6^ group and 10 from the 4 × 10^6^ group. 3, 4 and 4 patients from the lower, medium and higher dose completed the additional follow-up period, respectively (Table 2; Figure 2).

**Table 2:** ITT population that completed each follow-up period.

Follow-up	Group	No. ITT patients
Follow-up 1	1×10^6^ AdMSC/kg	10
	2×10^6^ AdMSC/kg	10
	4×10^6^ AdMSC/kg	8
	Placebo	10
	Total	38
Follow-up 2*	1×10^6^ AdMSC/kg	4
	2×10^6^ AdMSC/kg	0
	4×10^6^ AdMSC/kg	3
	Total	7
Additional follow-up	1×10^6^ AdMSC/kg	3
	2×10^6^ AdMSC/kg	4
	4×10^6^ AdMSC/kg	4
	Total	11

*Only placebo group.

In Table 3 the demographic and clinical baseline characteristics of the participants are shown, with all groups being similar and not showing significant differences (consult Supplementary Figure S1 for baseline ALSFRS-R scores).

**Table 3:** Baseline characteristics of the participants.

Characteristics	1 × 10^6^ AdMSC/kg (*n* = 10)	2 × 10^6^ AdMSC/kg (*n* = 12)	4 × 10^6^ AdMSC/kg (*n* = 8)	Placebo (*n* = 10)
Sex—no. (%)				
Female	5 (50.00)	7 (58.33)	4 (50.0)	3 (30.00)
Male	5 (50.00)	5 (41.67)	4 (50.00)	7 (70.00)
Mean age—years (SD)	52.94 (5.34)	55.45 (9.16)	49.95 (4.84)	49.20 (14.55)
Mean weight—kg (SD)	67.27 (10.89)	69.29 (15.60)	66.63 (14.32)	69.44 (14.00)
Mean number of previous pathologies—no. (SD)	3.30 (3.23)	5.92 (2.39)	3.38 (1.69)	3.40 (2.55)
Mean age at ALS symptoms onset—years (SD)	51.42 (5.37)	53.80 (9.40)	48.52 (4.37)	47.25 (14.92)
Mean months since beginning of ALS to inclusion—no. (SD)	18.20 (7.81)	19.86 (9.64)	17.25 (8.99)	23.36 (9.09)
Type of ALS involvement—no. (%)				
Pyramidal	9 (90.00)	8 (66.67)	6 (75.00)	7 (70.00)
Bulbar	1 (10.00)	4 (33.33)	2 (25.00)	3 (30.00)
Mean number of ALS symptoms—no. (SD)	3.80 (1.81)	4.92 (2.99)	3.38 (3.50)	5.30 (1.89)
Severity of ALS symptoms—no. (%)				
Mild	19 (50.00)	23 (38.89)	6 (22.22)	26 (49.06)
Moderate	16 (42.11)	33 (55.93)	20 (74.07)	13 (24.53)
Severe	3 (7.89)	3 (5.08)	1 (3.70)	14 (26.42)
Mean FVC—% (SD)	80.96 (17.49)	84.76 (16.73)	84.24 (12.43)	75.31 (17.97)

SD, standard deviation.

### 3.2 Safety results

Most frequent Adverse Events (AEs) reported during the study were dysphagia (5.48%), muscular weakness (4.44%), respiratory failure (4.18%), headache (3.92%) and dysarthria (3.13%).

A total of 210 AEs occurred during the first follow-up period: 52 (24.76%) in the placebo group (*n* = 10) and a total of 158 (72.24%) in the three groups treated with stem cell therapy (*n* = 30). Out of those, 55 (34.81%) occurred in the lower dose group, 55 (34.81%) in the medium dose group and 48 (30.38%) in the higher dose group. Most frequent AEs were muscle weakness (5.88% of events), headache (5.43%), dysphagia (3.62%) and pyrexia (3.17%). 12 of the 158 AEs were considered related to the treatment administered: 1 case of phlebitis and 1 of phlebitis superficial in 1 × 10^6^ AdMSC/kg group; 1 case of headache in 2 × 10^6^ AdMSC/kg group; 2 cases of headache,1 pyrexia, 3 phlebitis and 1 thrombophlebitis in 4 × 10^6^ AdMSC/kg group and 1 case of headache in placebo group. A total of 11 SAEs were also reported during this period (Table 4), 1 of them in the placebo and the rest in AdMSC treatment groups (1 in lower dose, 6 in medium dose and 3 in higher dose). A case of deep vein thrombosis in a patient that received the higher dose of AdMSC group, that arose 6 days after the administration, was reported as a serious adverse reaction and was considered related to the administration procedure.

**Table 4:** Adverse events and serious adverse events during follow-up 1.

Number of events	Placebo		Treatment		Treatment					
					1 × 10^6^ AdMSC/kg		2 × 10^6^ AdMSC/kg		4 × 10^6^ AdMSC/kg	
	*N*	%	*N*	%	*N*	%	*N*	%	*N*	%
Adverse events (AEs)	52	24.76	158	72.24	55	34.81	55	34.81	48	30.38
Serious adverse events (SAEs)	1	9.09	10	90.91	1	10.00	6	60.00	3	30.00
Adverse reactions	0	0.00	1	100	0	0.00	0	0.00	1*	100
AES probably or definitively related to the treatment	1	8.33	11	91.67	2	18.18	1	9.09	7	63.64
SAEs probably or definitively related to the treatment	0	0.00	1	100	0	0.00	0	0.00	1	100

Percentages of placebo versus treated calculated over the total number of events in each category. Percentages of each treated group calculated over the total number of events in each category of the total number of events occurring in treated patients. *Serious adverse reaction (SAR) related to the infusion procedure.

In the second follow-up period, a total of 35 AEs occurred (Table 5): 11 AEs (31.43%) occurred in the lower dose group, 7 (20.00%) in the medium dose group and 17 (48.57%) in the higher dose group. Most frequent AEs were dysarthria, dysphagia, dyspnea, headache, hypertension, muscle weakness, salivary hypersecretion, deep vein thrombosis and respiratory tract infection (5.00% each). 4 out of the 35 AEs were related to the treatment administered: 1 case of headache in 1 × 10^6^ AdMSC/kg group; 1 case of procedural pain in 2 × 10^6^ AdMSC/kg group; and 1 case of headache and 1 of phlebitis superficial in 4 × 10^6^ AdMSC/kg group. A total of 5 SAEs were reported during this period, 3 in the medium dose of AdMSC group and 2 in the higher dose. A case of deep vein thrombosis in a patient that received the medium dose of AdMSC group was reported the day of the administration as a serious adverse reaction and was considered related to the administration procedure.

**Table 5:** Adverse events and serious adverse events during follow-up 2.

Number of events	Treatment					
	1 × 10^6^ AdMSC/kg		2 × 10^6^ AdMSC/kg		4 × 10^6^ AdMSC/kg	
	*N*	%	*N*	%	*N*	%
Adverse events (AEs)	11	31.43	7	20.00	17	48.57
Serious adverse events (SAEs)	0	0.00	3*	60.00	2	40.00
Adverse reactions	0	0.00	1	100	0	0.00
AEs probably or definitively related to the treatment	1	25.00	1	25.00	2	50.00
SAEs probably or definitively related to the treatment	0	0.00	1	100	0	0.00

Percentages of each treated group calculated over the total number of events in each category of the total number of events occurring in treated patients. *One of the SAEs was a SAR.

During the additional follow-up, the most frequent AEs were respiratory failure (11.48%), dysphagia (9.02%), nasopharyngitis (6.56%), pneumonia (4.92%), dysarthria (4.10%), constipation (3.28%) and evolution of ALS (3.28%). A total of 84 AEs were reported during this period: 33 (39.29%) in the lower dose of AdMSC group, 27 (32.14%) in the second dose group and 24 (28.57%) in the higher dose group (Table 6); *n*. A total of 38 SAEs were reported, 14 (36.64%) in the lower dose of AdMSC group, 14 (36.64%) in the medium dose group and 10 (26.32%) in the higher dose group. None of the AEs and SAEs occurred during this period were related to the treatment administered.

**Table 6:** Adverse events and serious adverse events during additional follow-up.

Number of events	Treatment					
	1 × 10^6^ AdMSC/kg		2 × 10^6^ AdMSC/kg		4 × 10^6^ AdMSC/kg	
	*N*	%	*N*	%	*N*	%
Adverse events (AEs)	33	39.29	27	32.14	24	28.57
Serious adverse events (SAEs)	14	36.84	14	36.84	10	26.32
AEs probably or definitively related to the treatment	0	0.00	0	0.00	0	0.00
SAEs probably or definitively related to the treatment	0	0.00	0	0.00	0	0.00

Percentages of each treatment group calculated over the total number of events in each group in the additional follow-up period.

Aside from the analysis of AEs during the first follow-up period—where patients in the placebo group tended to have non-treatment-related events, while patients on higher doses of AdMSC had a somewhat higher proportion of likely treatment-related events than the other groups (*p* = 0.015)—no relationship between AEs or SAEs and treatment administered was found.

A total of 23 patients (57.5%) died during the study: 7 in 1 × 10^6^ AdMSC/kg group (additional follow-up), 8 in 2 × 10^6^ AdMSC/kg group (2 in follow-up 1, 6 in follow-up 2), 3 in 4 × 10^6^ AdMSC/kg group (additional follow-up) and 5 in placebo group (1 in follow-up 2, 4 in additional follow-up). Many of the patients died as a consequence of respiratory failure (*n* = 16) associated with the progression of ALS. The second leading cause of death in patients was pneumonia (*n* = 2).

### 3.3 Feasibility results

Extraction was successfully performed in all the patients, even though some complications arose during the procedure in four patients (post procedural hematoma (*n* = 1) in 2 × 10^4^ AdMSC/kg group, procedural pain (*n* = 1) in 4 × 10^6^ AdMSC/kg group, drainage (*n* = 1) and seroma (*n* = 1) in placebo group).

Infusion was successfully performed in all the patients both in the first and the second infusion procedures. However, there were complications during the administration procedure, in most cases events of phlebitis and thrombosis. During the first follow-up period, 4 complications were reported in the medium dose of AdMSC group [phlebitis (*n* = 1), thrombophlebitis (*n* = 1), phlebitis superficial (*n* = 1) and pyrexia (*n* = 1)], whereas 2 were reported in the higher dose group [thrombophlebitis (*n* = 1) and deep vein thrombosis (*n* = 1)]. During the second follow-up period, in which patients that initially received placebo were randomized to receive treatment with AdMSC, 1 event of headache was reported in the lower dose group, 2 complications in the medium dose group [deep vein thrombosis (*n* = 1) and procedural pain (*n* = 1)] and 4 in the higher dose group [hypertension (*n* = 1), phlebitis superficial (*n* = 1), abdominal pain (*n* = 1) and headache (*n* = 1)].

### 3.4 Efficacy results

Changes in the disease progression were measured through changes in total punctuation in ALSFRS-R scale. A certain decrease in the scale scores over time was observed and patients tended to worsen compared to baseline state, showing the degenerative pattern of the disease, with no differences between groups nor different pattern between groups (Supplementary Figure S2). The delay in receiving stem cell therapy did not appear to affect the disease progression in any way (Supplementary Figures S3, S4). A decrease in all the domains over time was observed in the first 6 months, except from the respiratory domain, whose scores remained high regardless of the randomization group.

Secondly, a reduction in muscle strength through MRC scale was observed in the scores after 6 months (*p* < 0.001) with no differences between groups.

A certain decrease in FVC, measured through spirometry, was observed as time goes by, with no differences between groups. The pattern of decline was neither observed to be different depending on the group (Supplementary Figure S5).

The analysis of variations in muscle mass by MRI was performed individually per patient, comparing the same segment and the same limb in each patient. In general, it was observed that patients tended to worsen after 6 months as a consequence of disease progression.

On the other hand, changes in extremities circumference were assessed in right and left arm, forearm, thigh and leg. No strong variations in mean extremity circumference were observed, although at 6 months the variability seemed to increase. Nevertheless, the high percentage of missing values prevented the execution of an inferential analysis.

Regarding neurophysiological parameters, a decrease in the values of the neurophysiological index was observed after 6 months with a similar pattern in all groups (Supplementary Figure S6). In addition, changes in MUNE values, as well as the amplitude and excitability threshold of MEPs, were individually analyzed. A general decrease in the values 6 months since baseline was observed; however, some individual cases showed a slight favorable change for these parameters.

Following the analysis of neuropsychological parameters through WAIS-III scale, the patients in the group with the highest dose of AdMSC had hardly any data on these variables for evaluation after 6 months, so it was not possible to study whether there were significant differences between groups and time.

In relation to changes in quality of life, measured through the SIP, there was a high percentage of missing values. In general, patients scored low, indicating that there was no great impact of the disease in their quality of life. In some isolated cases there are very high values, but in these patients there was no information available (in baseline or after 6 months) to study whether changes occurred. During the first follow-up period, the results showed that the scores in sleep and rest (*p* = 0.038) and in communication (*p* = 0.036) were somewhat higher after 6 months, regardless of the treatment. In nutrition, there were significant differences associated with the passing of time (*p* = 0.004) and group (*p* = 0.039). However, given that there are discordant data in some of the patients, these results should be handled with caution.

Spasticity was assessed using the Ashworth scale. Muscle tone was generally normal in most patients, although it was observed that spasticity tended to be more prevalent in the knees and ankles in comparison to the rest of the limbs. In the first follow-up period, it was observed that spasticity increased significantly over time in the right elbow (*p* = 0.005), right knee (*p* < 0.001), left knee (p < 0.001), right ankle (*p* = 0.010) and left ankle (*p* = 0.047), possibly as a consequence of the degenerative effect of ALS. No differences between groups or group-time interaction were found, except in the case of the left elbow, where the increase in spasticity was somewhat more pronounced in the higher dose of AdMSC group (*p* = 0.026). When analyzing the changes by dose of stem cell therapy received, the results showed that spasticity increased significantly with time in the right elbow (*p* = 0.030), right knee (*p* = 0.003) and left knee (*p* = 0.020), possibly as a consequence of the degenerative effect of ALS. No between-group differences or dose-time interaction were found. Although there were signs of improvement in many patients when individually analyzed, in most cases it was an absence of change from baseline rather than a decrease of one or more points on the Ashworth scale. Statistical analysis of the percentage of clinical improvement showed no difference between groups.

The analysis of pain rating through the VAS scale showed most patients had a moderately high pain rating that tended to increase over time, becoming moderately elevated at 6 months of follow-up (*p* = 0.014), with no significant differences between groups (*p* = 0.488) or group-time interaction effect (*p* = 0.187). During the second follow-up period, pain ratings continued increasing over time in all groups. When analyzing the improvement in the score of the scale, it was observed that in many of the patients there tended to be some worsening in pain perception, although no difference between treatment groups was found.

Regarding the McGill questionnaire, an increase in the ratings over time of the PRI (*p* = 0.037) and the number of words chosen (*p* = 0.038) was found during the first 6 months, with no differences between groups or group-time interaction.

As for the need for gastrostomy, when analyzed based on the initial randomization group, it was observed that in the group with the lowest dose of AdMSC no patient needed gastrostomy. In the rest of the groups, they did need it with a greater probability than in the lower stem cell group. However, when comparing the probability of needing gastrostomy within each group, in all groups except the placebo group there was a higher proportion of patients who did not need gastrostomy (100% (lower dose of AdMSC), 66.67% (medium dose of AdMSC) and 50% (higher dose of AdMSC) versus 40% (placebo), *p* = 0.030). Nonetheless, when analyzed based on the dose of AdMSC received, the probability of needing gastrostomy did not differ significantly between treatment groups (*p* = 0.107).

**Figure 3:**
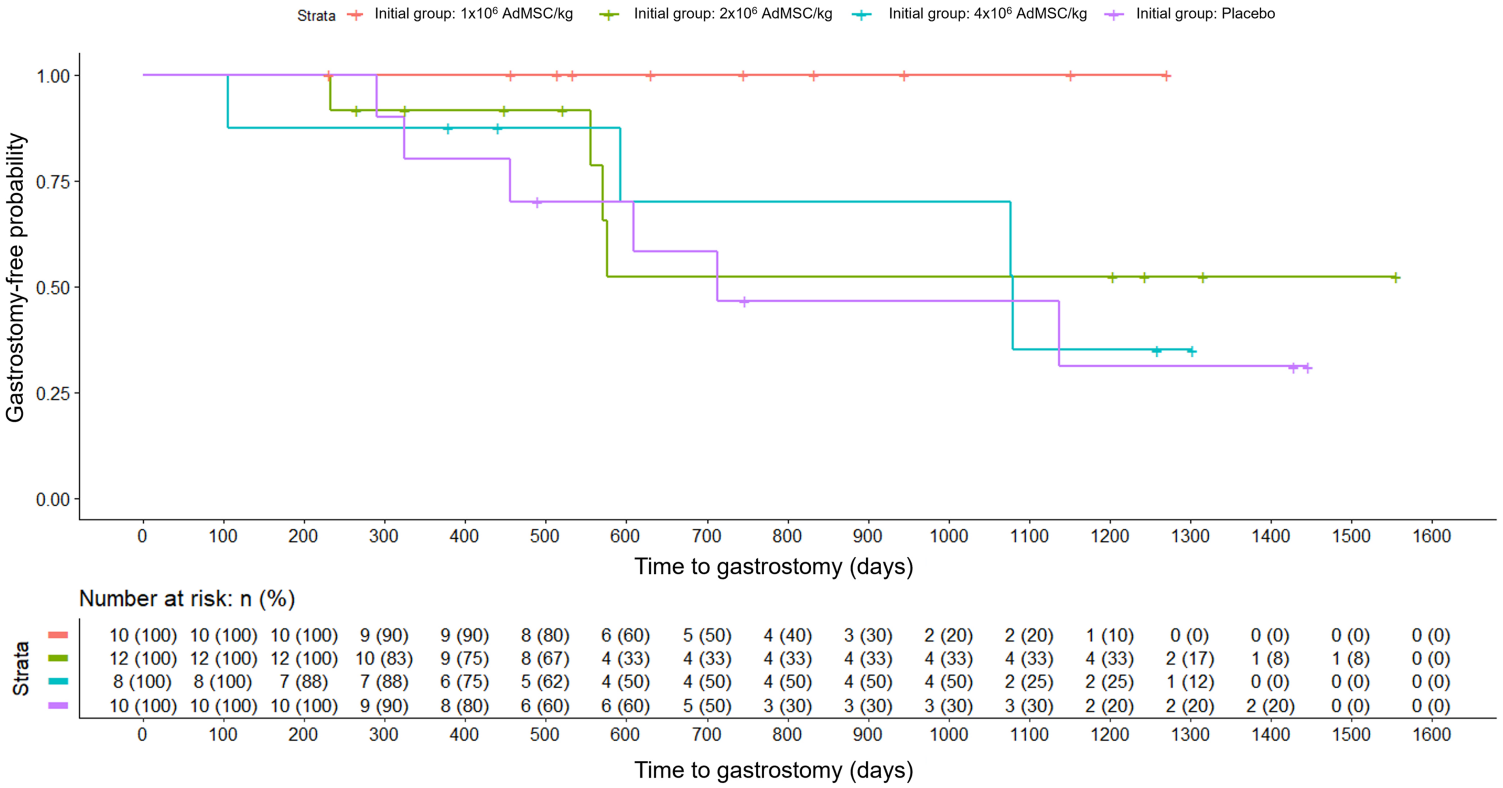
Time to gastrostomy by initial randomization group (Kaplan–Meier). Groups: placebo; AdMSC 1 × 10^6^, 2 × 10^6^, 4 × 10^6^ cells/kg; risk table shown. Cox model Likelihood Ratio Test p = 0.040 (nominal; interpret cautiously).

**Figure 4:**
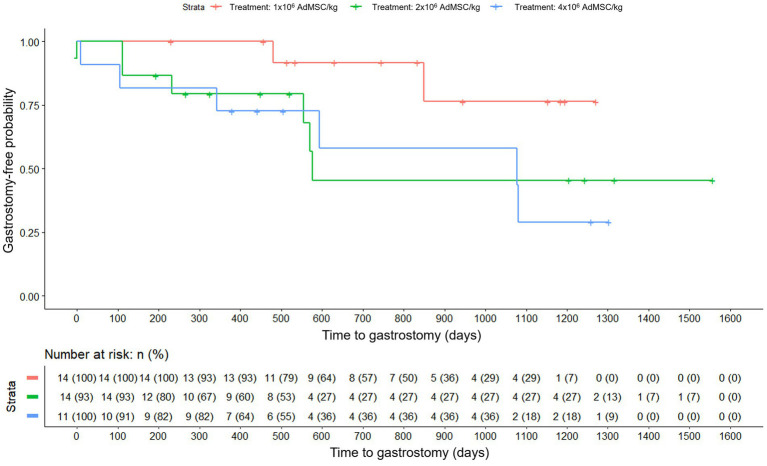
Time to gastrostomy by dose actually received (Kaplan–Meier). Groups: AdMSC 1 × 10^6^, 2 × 10^6^, 4 × 10^6^ cells/kg; risk table shown. Cox model Likelihood Ratio Test p = 0.090.

When analyzing the time to gastrostomy based on the initial randomization group, the survival curves differed (Figure 3) and Cox regression indicated a nominally significant difference (Likelihood Ratio Test, *p* = 0.040). By contrast, the analysis by dose of AdMSC actually received, was not significant (Likelihood Ratio Test, *p* = 0.090) (Figure 4). Given the small sample and multiple exploratory comparisons, these findings should be interpreted cautiously.

Only two patients needed permanent assisted ventilation (PAV), one from the lower dose of AdMSC group, and another from the medium dose of AdMSC group. In both cases, PAV was established during an event of respiratory failure in the additional follow-up period. Both patients also needed tracheostomy to be performed during the course of those events.

**Figure 5:**
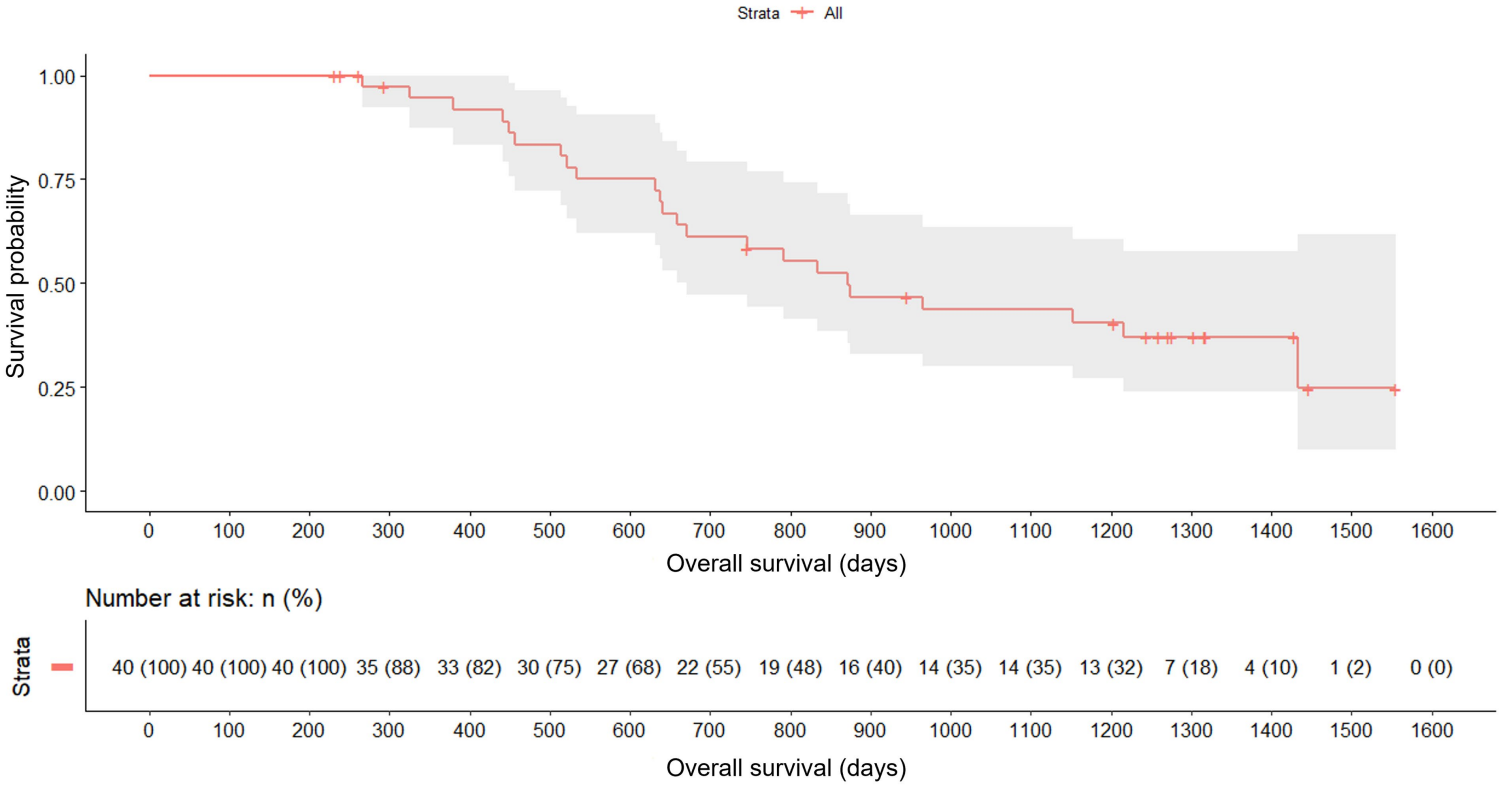
Overall survival for the full cohort since baseline (Kaplan–Meier) with 95% CIs and number-at-risk table.

Regarding the overall survival, 57.5% (23/40) of the patients died during the study. 2 patients died during the first 6 months, all from the medium dose of AdMSC group. Deaths during the study were clustered between 13 and 32 months, during the additional follow-up. Taking the entire follow-up period as the analysis time, the median survival was 872 days. No ITT patient died until approximately 200 days since baseline, with a sharp drop in the survival curve between day 400 and day 1,000. In addition, there was a high rate of abandonment between days 1,200 and 1,300 (Figure 5).

**Figure 6:**
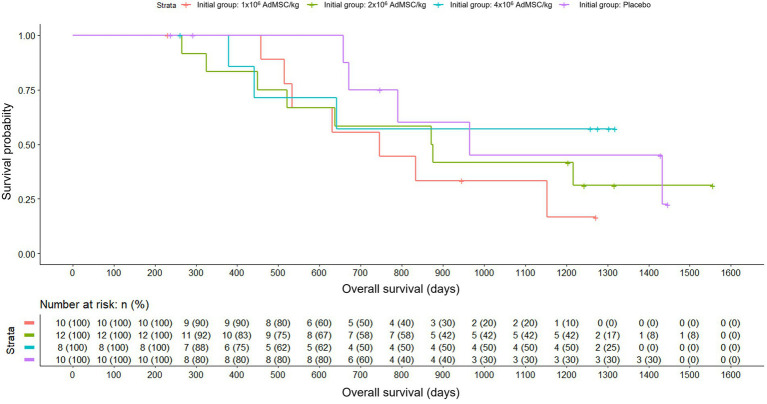
Overall survival by initial randomization group: placebo; AdMSC 1 × 10^6^, 2 × 10^6^, 4 × 10^6^ cells/kg (Kaplan–Meier) with number-at-risk table.

Analyzing the overall survival in the initial randomization group, the results showed that the mean survival rates were somewhat higher in the placebo group and in the group with a higher dose of AdMSC. However, when applying Cox regression, there were no statistically significant differences between groups (Likelihood Ratio Test, *p* = 0.600). In the group with the highest dose of AdMSC, less than 50% of the patients died and deaths in this group appeared between days 400 and 600 approximately. In all groups except the medium dose group, there were no deaths during the first 6 months. The first death in the placebo group appeared later than in the other groups. The probability of death increased for all groups between days 650 and 1,000, which was where the survival curves had the steepest slope (Figure 6).

Overall survival was also analyzed based on the dose of stem cell therapy received, during the active treatment period (time was counted from the time when all patients have received their dose of stem cell therapy) and since baseline.

**Figure 7:**
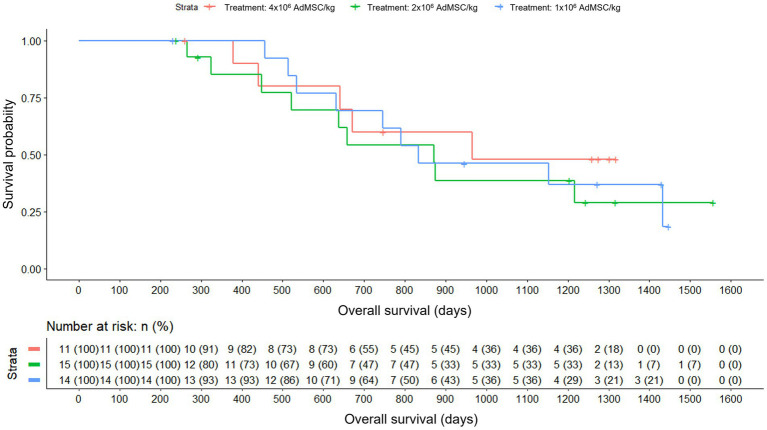
Overall survival by dose actually received: AdMSC 1 × 10^6^, 2 × 10^6^, 4 × 10^6^ cells/kg (Kaplan–Meier) with number-at-risk table; Cox Likelihood Ratio Test p = 0.800.

When the active treatment period is analyzed, it appears that the mean survival is somewhat higher in the group with the highest dose of stem cell therapy, in addition to the fact that the patients in this group are less likely to die. The slope was steeper in the lower cell product dose group, especially between days 500 and 850, while in the other two groups the slope was somewhat more progressive. However, no differences between groups were found when applying Cox regression (Likelihood Ratio Test, *p* = 0.800) (Figure 7).

When analyzed since baseline, the probability of survival and its decrease appeared to be very similar and progressive among the different doses, except in the lower dose group, where deaths were more prevalent between days 500 and 800. In the higher dose group, there were fewer deaths and the curve seemed to have a lower slope, while the first case of death in the lower dose group appeared later. Nonetheless, no statistically significant differences between groups were found (Likelihood Ratio Test, *p* = 0.800).

Taking into consideration the region of origin of ALS symptoms, 50% of the patients with bulbar involvement and 60% of the patients with pyramidal involvement died. Although survival was somewhat higher in the case of bulbar involvement, the differences were not statistically significant (Likelihood Ratio Test, *p* = 0.500), possibly due to the small sample size of the group of patients with bulbar involvement.

Lastly, the functional analysis of the immune response could not be performed due to a problem in the labeling and storage of the blood samples. However, 1H-NMR spectra were obtained on the available samples. The 18 samples belonged to 9 patients who received AdMSC and completed the first follow-up (each patient had two samples: before infusion and after infusion). The statistical analysis revealed a significant decrease in histidine (*p* = 0.0405980) and lysine (0.0495058) concentrations in CSF after treatment at a 95% confidence interval. However, since the number of control samples was not sufficient for inclusion in the analysis, it could not be ruled out that these differences were due to disease progression rather than the effects of treatment with AdMSC.

## 4 Discussion

This clinical trial showed that treatment with AdMSC is feasible and safe in patients with ALS in the short and long term. Both the extraction and administration procedures could be successfully performed in all the patients included.

Despite the high number of adverse events, most of them were attributable to the natural progression of the pathology. No relevant relationship with the treatment with AdMSC was found. The occurrence of new neurological effects not attributable to the natural progression of ALS was also not observed. The main safety problem encountered were problems related to the infusion of the stem cell therapy, specifically events of phlebitis and thrombosis, suggesting that the infusion rate and the administration procedures should be reevaluated to minimize the risk of occurrence of these events.

Regarding the evaluation of efficacy of AdMSC, no sign or trend of efficacy was observed in any of the variables analyzed. When comparing the results of the first follow-up with those of the second, the results obtained were very similar. The efficacy results in both follow-ups highlight the degenerative factor of the disease in the short and long term. It is observed that the delay in receiving treatment with AdMSC (patients initially randomized to the placebo group) does not affect in any way the progression of the disease. Comparison of results between first and second follow-up periods does not indicate that the delay in the administration of AdMSC product affected the progression of ALS. However, the high number of missing values non-valuable data in many of the variables make drawing conclusions difficult. In certain variables, such as for FVC% and WAIS-III, values were missing or not valid because disease progression and the motor limitation of the patients prevented the accurate performance of the evaluations in many cases. Regarding the metabolomic analysis, even though the statistical analysis revealed a significant decrease in histidine and lysine concentrations in CSF, the results have low statistical power due to the reduced sample size and the small variation in metabolite concentrations between groups. Furthermore, no control samples were included in the analysis because of their small number, hence it cannot be ruled out that these differences were due to disease progression rather than the effects of treatment with AdMSC. Indeed, histamine, synthesized from histidine, has been associated with neuroprotective effects slowing disease progression in animal models of ALS (64). On the other hand, previous studies suggest that altered lysine transport, potentially mediated by reduced cationic amino acid transporter-1 (CAT-1) expression, contributes to the pathophysiology of ALS (65). Therefore, the decreased levels of histidine and lysine found in the study are likely attributable to disease progression. Nevertheless, further studies with larger cohorts are necessary to elucidate the potential involvement of these metabolites in disease progression or response to treatment.

It is important to mention that efficacy has been assessed in a very comprehensive manner, through different clinical variables that cover neurological and neuromuscular degeneration that usually accompany the progression of ALS disease. Additionally, paraclinical variables (such as muscle mass measured by MRI, neurophysiological index, MUNE, MEP, neuropsychological tests and quality of life measure), as surrogate markers of the clinical variable, were used for assessing the degree of motor neuron involvement. Nonetheless, the need to perform a high number of tests on patients in a delicate condition may had led to a poor data collection in certain cases. Furthermore, the fact of having performed numerous analyses led to an increase in the type I error, making any positive results that might be found less reliable.

Clinical experience with intravenous (IV) MSCs in ALS is limited but indicates feasibility and an acceptable safety profile, with inconclusive efficacy. An open-label phase I comparing IV vs. intrathecal autologous BM-MSCs reported no safety signals but ALSFRS-R and FVC declined at expected rates in both routes (66). A single-center, prospective study of allogeneic IV Ad-MSCs likewise found IV delivery safe and feasible without definitive evidence of slowed progression (67). Randomized programs have largely emphasized non-IV routes (68), underscoring that route, dose density, and patient selection remain open questions.

More than half of the patients (57.5%) died during the course of the study, mostly due to respiratory failure or pneumonia, most probably due to the natural evolution of the disease. However, only two patients required both PAV and a tracheostomy procedure. Since the start of the study in 2014 and its development, multi-disciplinary units in hospitals have considerably progressed and care guidelines have been updated with the knowledge gained over the past 20 years. The considerations and establishment of mechanical invasive ventilation through tracheostomy have been refined and evolved since the study started, hence impacting the standard care of patients, making it difficult to extrapolate these results to the present time.

The study has some limitations. First, the sample size, selected based on clinical criteria, was relatively small to allow conclusions related to efficacy results to be drawn. However, it should be taken into consideration that the present study was a phase I/II clinical whose main objective was to demonstrate the safety of the treatment, which was successfully proved. Secondly, the high number of missing values likely contributed to the lack of statistically significant differences between treatments. On the other hand, even though it could not be performed, the functional analysis of the immune response could have provided essential data to elucidate how the infusion of mesenchymal stem cells influences the immune status of patients with ALS and the development of the disease. Regarding the metabolomic analysis, it should be noted that the results have low statistical power, making it challenging to draw reliable conclusions. In addition, the performance of the feasibility analysis carried some limitations because the study design did not consider differentiating between extraction or infusion related AEs during data collection, and these AEs were reported in many cases as related to the treatment with AdMSC.

The results obtained in this study are consistent with the ones obtained in other clinical trials using similar doses of MSC, where safety was demonstrated and efficacy values were inconclusive, due to not reaching statistical significance (44, 69–71), providing only a slight clinical benefit (39, 40, 68) or lacking a control group (45, 72). It must be noted that in some of the recent studies, although the doses administered were similar, repeated injections regimes were chosen, administering 2 monthly injections (41, 42), 1–3 monthly injections (70), 3 injections every 2 months (71) or 1–4 injections at intervals of 3–6 months (39). However, repeated administrations did not appear to provide better efficacy results, apart from the study performed by Oh et al. (42). The optimization of dose frequency and the administration of intravenous and intrathecal combinations are encouraged to be explored. Larger studies with an increased sample size, different doses and route of administration, or combination of routes, repeated dosing or larger duration and comprehensive assessment of immunological effects would be needed to analyze the efficacy of AdMSC in the treatment of ALS.

In summary, a single IV infusion of autologous AdMSCs at 1–4 × 10^6^ cells/kg was feasible and showed an acceptable safety profile, with line-related phlebitis/thrombosis as the main procedural risks. Efficacy endpoints showed no statistically significant benefit versus placebo, and any numerical differences across doses were inconsistent and underpowered for inference. These results align with prior IV-MSC experiences in ALS—safety with inconclusive efficacy—and could, although doubtfully, support future studies that test repeated dosing and/or alternative or combined routes, refine infusion procedures to mitigate vascular events, and incorporate pharmacodynamic biomarkers to verify target engagement. Until such data emerge, IV AdMSC therapy should be regarded as investigational with reassuring safety but unproven efficacy.

## Data Availability

The original contributions presented in the study are included in the article/Supplementary material, further inquiries can be directed to the corresponding author/s.

## Glossary

AE: Adverse Event
AdMSC/AdMSCs: Adipose-derived Mesenchymal Stem Cell/Cells
ALS: Amyotrophic Lateral Sclerosis
bFGF: Basic Fibroblast Growth Factor
CAT-1: Cationic Amino Acid Transporter-1
CSF: Cerebrospinal Fluid
FVC: Forced Vital Capacity
GDNF: Glial-Derived Neurotrophic Factor
HRMAS: High Resolution Magic Angle Spinning
ICH: International Council for Harmonisation of Technical Requirements for Pharmaceuticals for Human Use
IgG: Immunoglobulin G
ITT: Intention To Treat
IV: Intravenous
mRNA: Messenger Ribonucleic Acid
MMT: Manual Muscle Testing
MRC: Medical Research Council
MRI: Magnetic Resonance Imaging
MSC: Mesenchymal stem cells
MUNE: Motor Unit Number Estimation
1H NMR: Proton Nuclear Magnetic Resonance
PAV: Permanent Assisted Ventilation
PBMC: Peripheral Blood Mononuclear Cell
PRI: Pain Rating Index
PPI: Present Pain Intensity
REML: Restricted Maximum Likelihood Method
SAR: Serious Adverse Reaction
SOD1: Superoxide Dismutase Type 1
SP: Safety Population
ssRNA: Single Strand Ribonucleic Acid
SUSAR: Suspected Unexpected Serious Adverse Reaction
TLR: Toll-like receptor
VAS: Visual Analogue Scale
WAIS: Wechsler Adult Intelligence Scale

## References

[1] Taylor JP Brown RH Cleveland DW. Decoding ALS: from genes to mechanism. Nature. (2016) 539:197–206. doi: 10.1038/nature20413, PMID: 27830784 PMC5585017

[2] Brown RH Al-Chalabi A. Amyotrophic lateral sclerosis. N Engl J Med. (2017) 377:162–72. doi: 10.1056/NEJMra1603471, PMID: Longo DL, editor 28700839

[3] Chia R Chiò A Traynor BJ. Novel genes associated with amyotrophic lateral sclerosis: diagnostic and clinical implications. Lancet Neurol. (2018) 17:94–102. doi: 10.1016/S1474-4422(17)30401-5, PMID: 29154141 PMC5901717

[4] Mejzini R Flynn LL Pitout IL Fletcher S Wilton SD Akkari PA. ALS genetics, mechanisms, and therapeutics: where are we now? Front Neurosci. (2019) 13:1310. doi: 10.3389/fnins.2019.01310, PMID: 31866818 PMC6909825

[5] Garnier M Camdessanché JP Cassereau J Codron P. From suspicion to diagnosis: exploration strategy for suspected amyotrophic lateral sclerosis. Ann Med. (2024) 56:2398199. doi: 10.1080/07853890.2024.2398199, PMID: 39233624 PMC11378651

[6] Mimic S Aru B Pehlivanoğlu C Sleiman H Andjus PR Yanıkkaya Demirel G. Immunology of amyotrophic lateral sclerosis – role of the innate and adaptive immunity. Front Neurosci. (2023) 17:1277399. doi: 10.3389/fnins.2023.1277399, PMID: 38105925 PMC10723830

[7] Marin B Boumédiene F Logroscino G Couratier P Babron MC Leutenegger AL . Variation in worldwide incidence of amyotrophic lateral sclerosis: a meta-analysis. Int J Epidemiol. (2017) 46:57–74. doi: 10.1093/ije/dyw061, PMID: 27185810 PMC5407171

[8] Xu L Liu T Liu L Yao X Chen L Fan D . Global variation in prevalence and incidence of amyotrophic lateral sclerosis: a systematic review and meta-analysis. J Neurol. (2020) 267:944–53. doi: 10.1007/s00415-019-09652-y, PMID: 31797084

[9] Centers for Disease Control and Prevention. National amyotrophic lateral sclerosis registry. (2025). Available online at: https://www.cdc.gov/als/dashboard/index.html (accessed April 25, 2025)

[10] Food and Drug Administration. Rare diseases at FDA. FDA; 2024. Available online at: https://www.fda.gov/patients/rare-diseases-fda (accessed April 25, 2025)

[11] European Parliament and the Council. Decision No 1295/1999/EC of the European Parliament and of the Council of 29 April 1999 adopting a programme of Community action on rare diseases within the framework for action in the field of public health (1999 to 2003) (1999). Available online at: https://eur-lex.europa.eu/eli/dec/1999/1295/oj/eng (accessed April 25, 2025)

[12] Saleh IA Zesiewicz T Xie Y Sullivan KL Miller AM Kuzmin-Nichols N . Evaluation of humoral immune response in adaptive immunity in ALS patients during disease progression. J Neuroimmunol. (2009) 215:96–101. doi: 10.1016/j.jneuroim.2009.07.011, PMID: 19682755

[13] Provinciali L Laurenzi MA Vesprini L Giovagnoli AR Bartocci C Montroni M . Immunity assessment in the early stages of amyotrophic lateral sclerosis: a study of virus antibodies and lymphocyte subsets. Acta Neurol Scand. (1988) 78:449–54. doi: 10.1111/j.1600-0404.1988.tb03686.x, PMID: 3265563

[14] Calvo A Moglia C Balma M Chio A. Involvement of immune response in the pathogenesis of amyotrophic lateral Sclerosis: a therapeutic opportunity? CNS Neurol Disord Drug Targets. (2010) 9:325–30. doi: 10.2174/187152710791292657, PMID: 20406178

[15] Kornberg AJ. Anti-GM1 ganglioside antibodies: their role in the diagnosis and pathogenesis of immune-mediated motor neuropathies. J Clin Neurosci. (2000) 7:191–4. doi: 10.1054/jocn.1999.0194, PMID: 10833614

[16] Van Blitterswijk M Gulati S Smoot E Jaffa M Maher N Hyman BT . Anti-superoxide dismutase antibodies are associated with survival in patients with sporadic amyotrophic lateral sclerosis. Amyotroph Lateral Scler. (2011) 12:430–8. doi: 10.3109/17482968.2011.585163, PMID: 22023190 PMC3446817

[17] Smith RG Siklos L Alexianu ME Engelhardt JI Mosier DR Colom L . Autoimmunity and ALS. Neurology. (1996) 47:S40–5. doi: 10.1212/wnl.47.4_suppl_2.40s, discussion S45-6 8858050

[18] La Bella V Goodman JC Appel SH. Increased CSF glutamate following injection of ALS immunoglobulins. Neurology. (1997) 48:1270–2. doi: 10.1212/WNL.48.5.1270, PMID: 9153455

[19] Henkel JS Engelhardt JI Siklós L Simpson EP Kim SH Pan T . Presence of dendritic cells, MCP-1, and activated microglia/macrophages in amyotrophic lateral sclerosis spinal cord tissue. Ann Neurol. (2004) 55:221–35. doi: 10.1002/ana.10805 14755726

[20] Kassa RM Mariotti R Bonaconsa M Bertini G Bentivoglio M. Gene, cell, and axon changes in the familial amyotrophic lateral Sclerosis mouse sensorimotor cortex. J Neuropathol Exp Neurol. (2009) 68:59–72. doi: 10.1097/NEN.0b013e3181922572, PMID: 19104445

[21] Barbeito AG Mesci P Boillée S. Motor neuron–immune interactions: the vicious circle of ALS. J Neural Transm. (2010) 117:981–1000. doi: 10.1007/s00702-010-0429-0, PMID: 20552235 PMC3511247

[22] Riley J Sweeney W Boulis N. Shifting the balance: cell-based therapeutics as modifiers of the amyotrophic lateral sclerosis–specific neuronal microenvironment. Neurosurg Focus. (2008) 24:E10. doi: 10.3171/FOC/2008/24/3-4/E9, PMID: 18341386

[23] Moisse K Strong MJ. Innate immunity in amyotrophic lateral sclerosis. Biochim Biophys Acta. (2006) 1762:1083–93. doi: 10.1016/j.bbadis.2006.03.001, PMID: 16624536

[24] Sabatelli M Madia F Conte A Luigetti M Zollino M Mancuso I . Natural history of young-adult amyotrophic lateral sclerosis. Neurology. (2008) 71:876–81. doi: 10.1212/01.wnl.0000312378.94737.45, PMID: 18596241

[25] Hinchcliffe M Smith A. Riluzole: real-world evidence supports significant extension of median survival times in patients with amyotrophic lateral sclerosis. Degener Neurol Neuromuscul Dis. (2017) 7:61–70. doi: 10.2147/DNND.S135748, PMID: 30050378 PMC6053101

[26] Uccelli A Pistoia V Moretta L. Mesenchymal stem cells: a new strategy for immunosuppression? Trends Immunol. (2007) 28:219–26. doi: 10.1016/j.it.2007.03.001, PMID: 17400510

[27] Uccelli A Moretta L Pistoia V. Mesenchymal stem cells in health and disease. Nat Rev Immunol. (2008) 8:726–36. doi: 10.1038/nri2395, PMID: 19172693

[28] Uccelli A Laroni A Freedman MS. Mesenchymal stem cells for the treatment of multiple sclerosis and other neurological diseases. Lancet Neurol. (2011) 10:649–56. doi: 10.1016/S1474-4422(11)70121-1, PMID: 21683930

[29] Kassis I Grigoriadis N Gowda-Kurkalli B Mizrachi-Kol R Ben-Hur T Slavin S . Neuroprotection and immunomodulation with mesenchymal stem cells in chronic experimental autoimmune encephalomyelitis. Arch Neurol. (2008) 65:753–61. doi: 10.1001/archneur.65.6.753, PMID: 18541795

[30] Kassis I Vaknin-Dembinsky A Karussis D. Bone marrow mesenchymal stem cells: agents of immunomodulation and neuroprotection. Curr Stem Cell Res Ther. (2011) 6:63–8. doi: 10.2174/157488811794480762, PMID: 20955154

[31] Zuk PA Zhu M Mizuno H Huang J Futrell JW Katz AJ . Multilineage cells from human adipose tissue: implications for cell-based therapies. Tissue Eng. (2001) 7:211–28. doi: 10.1089/107632701300062859, PMID: 11304456

[32] Han C Zhang L Song L Liu Y Zou W Piao H . Human adipose-derived mesenchymal stem cells: a better cell source for nervous system regeneration. Chin Med J. (2014) 127:329–37. doi: 10.3760/cma.j.issn.0366-6999.20120064, PMID: 24438624

[33] Ciervo Y Gatto N Allen C Grierson A Ferraiuolo L Mead RJ . Adipose-derived stem cells protect motor neurons and reduce glial activation in both in vitro and in vivo models of ALS. Mol Ther Methods Clin Dev. (2021) 21:413–33. doi: 10.1016/j.omtm.2021.03.017, PMID: 33869658 PMC8044387

[34] Marconi S Bonaconsa M Scambi I Squintani GM Rui W Turano E . Systemic treatment with adipose-derived mesenchymal stem cells ameliorates clinical and pathological features in the amyotrophic lateral sclerosis murine model. Neuroscience. (2013) 248:333–43. doi: 10.1016/j.neuroscience.2013.05.034, PMID: 23727509

[35] Řehořová M Vargová I Forostyak S Vacková I Turnovcová K Kupcová Skalníková H . A combination of intrathecal and intramuscular application of human mesenchymal stem cells partly reduces the activation of necroptosis in the spinal cord of SOD1G93A rats. Stem Cells Transl Med. (2019) 8:535–47. doi: 10.1002/sctm.18-0223, PMID: 30802001 PMC6525562

[36] Vercelli A Mereuta OM Garbossa D Muraca G Mareschi K Rustichelli D . Human mesenchymal stem cell transplantation extends survival, improves motor performance and decreases neuroinflammation in mouse model of amyotrophic lateral sclerosis. Neurobiol Dis. (2008) 31:395–405. doi: 10.1016/j.nbd.2008.05.016, PMID: 18586098

[37] Eggenhofer E Benseler V Kroemer A Popp FC Geissler EK Schlitt HJ . Mesenchymal stem cells are short-lived and do not migrate beyond the lungs after intravenous infusion. Front Immunol. (2012) 3:297. doi: 10.3389/fimmu.2012.00297, PMID: 23056000 PMC3458305

[38] Galipeau J Sensébé L. Mesenchymal stromal cells: clinical challenges and therapeutic opportunities. Cell Stem Cell. (2018) 22:824–33. doi: 10.1016/j.stem.2018.05.004, PMID: 29859173 PMC6434696

[39] Petrou P Kassis I Yaghmour NE Ginzberg A Karussis D. A phase II clinical trial with repeated intrathecal injections of autologous mesenchymal stem cells in patients with amyotrophic lateral sclerosis. Front Biosci (Landmark Ed). (2021) 26:693–706. doi: 10.52586/4980, PMID: 34719198

[40] Oh KW Moon C Kim HY Oh S i Park J Lee JH . Phase I trial of repeated intrathecal autologous bone marrow-derived mesenchymal stromal cells in amyotrophic lateral sclerosis. Stem Cells Transl Med. (2015) 4:590–7. doi: 10.5966/sctm.2014-0212, PMID: 25934946 PMC4449093

[41] Staff NP Madigan NN Morris J Jentoft M Sorenson EJ Butler G . Safety of intrathecal autologous adipose-derived mesenchymal stromal cells in patients with ALS. Neurology. (2016) 87:2230–4. doi: 10.1212/WNL.0000000000003359, PMID: 27784774 PMC5123559

[42] Oh KW Noh M Kwon M Kim HY Oh S Park J . Repeated intrathecal mesenchymal stem cells for amyotrophic lateral sclerosis. Ann Neurol. (2018) 84:361–73. doi: 10.1002/ana.25302, PMID: 30048006 PMC6175096

[43] Mazzini L Mareschi K Ferrero I Vassallo E Oliveri G Nasuelli N . Stem cell treatment in amyotrophic lateral Sclerosis. J Neurol Sci. (2008) 265:78–83. doi: 10.1016/j.jns.2007.05.016, PMID: 17582439

[44] Geijo-Barrientos E Pastore-Olmedo C De Mingo P Blanquer M Gómez Espuch J Iniesta F . Intramuscular injection of bone marrow stem cells in amyotrophic lateral Sclerosis patients: a randomized clinical trial. Front Neurosci. (2020) 14:195. doi: 10.3389/fnins.2020.00195, PMID: 32265627 PMC7105864

[45] Karussis D Karageorgiou C Vaknin-Dembinsky A Gowda-Kurkalli B Gomori JM Kassis I . Safety and immunological effects of mesenchymal stem cell transplantation in patients with multiple sclerosis and amyotrophic lateral sclerosis. Arch Neurol. (2010) 67:1178–94. 10.1001/archneurol.2010.248 PMC3036569 20937945

[46] Connick P Kolappan M Crawley C Webber DJ Patani R Michell AW . Autologous mesenchymal stem cells for the treatment of secondary progressive multiple sclerosis: an open-label phase 2a proof-of-concept study. Lancet Neurol. (2012) 11:150–6. doi: 10.1016/S1474-4422(11)70305-2, PMID: 22236384 PMC3279697

[47] Cohen JA Imrey PB Planchon SM Bermel RA Fisher E Fox RJ . Pilot trial of intravenous autologous culture-expanded mesenchymal stem cell transplantation in multiple sclerosis. Mult Scler J. (2018) 24:501–11. doi: 10.1177/1352458517703802, PMID: 28381130 PMC5623598

[48] Dahbour S Jamali F Alhattab D Al-Radaideh A Ababneh O Al-Ryalat N . Mesenchymal stem cells and conditioned media in the treatment of multiple sclerosis patients: clinical, ophthalmological and radiological assessments of safety and efficacy. CNS Neurosci Ther. (2017) 23:866–74. doi: 10.1111/cns.12759, PMID: 28961381 PMC5698713

[49] Kurtzberg J Abdel-Azim H Carpenter P Chaudhury S Horn B Mahadeo K . A phase 3, single-arm, prospective study of Remestemcel-L, ex vivo culture-expanded adult human mesenchymal stromal cells for the treatment of pediatric patients who failed to respond to steroid treatment for acute graft-versus-host disease. Biol Blood Marrow Transplant. (2020) 26:845–54. 32018062 10.1016/j.bbmt.2020.01.018 PMC8322819

[50] Brooks BR Miller RG Swash M Munsat TL. El Escorial revisited: revised criteria for the diagnosis of amyotrophic lateral sclerosis. Amyotroph Lateral Scler Other Motor Neuron Disord. (2000) 1:293–9. doi: 10.1080/146608200300079536, PMID: 11464847

[51] Frudinger A Pfeifer J Paede J Kolovetsiou-Kreiner V Marksteiner R Halligan S. Autologous skeletal-muscle-derived cell injection for anal incontinence due to obstetric trauma: a 5-year follow-up of an initial study of 10 patients. Color Dis. (2015) 17:794–801. doi: 10.1111/codi.12947, PMID: 25773013

[52] the Stem Cells in Multiple Sclerosis (STEMS) Consensus Group Martino G Franklin RJM Van Evercooren AB Kerr DA. Stem cell transplantation in multiple sclerosis: current status and future prospects. Nat Rev Neurol. (2010) 6:247–55. doi: 10.1038/nrneurol.2010.35, 20404843

[53] Freedman MS Bar-Or A Atkins HL Karussis D Frassoni F Lazarus H . The therapeutic potential of mesenchymal stem cell transplantation as a treatment for multiple sclerosis: consensus report of the international MSCT study group. Mult Scler J. (2010) 16:503–10. doi: 10.1177/1352458509359727, PMID: 20086020

[54] Cedarbaum JM Stambler N Malta E Fuller C Hilt D Thurmond B . The ALSFRS-R: a revised ALS functional rating scale that incorporates assessments of respiratory function. J Neurol Sci. (1999) 169:13–21. doi: 10.1016/S0022-510X(99)00210-5, PMID: 10540002

[55] Castrillo-Viguera C Grasso DL Simpson E Shefner J Cudkowicz ME. Clinical significance in the change of decline in ALSFRS-R. Amyotroph Lateral Scler. (2010) 11:178–80. doi: 10.3109/17482960903093710, PMID: 19634063

[56] Compston A. Aids to the investigation of peripheral nerve injuries. Medical Research Council: nerve injuries research committee. His majesty’s stationery office: 1942; pp. 48 (iii) and 74 figures and 7 diagrams; with aids to the examination of the peripheral nervous system. By Michael O’Brien for the Guarantors of Brain. Saunders Elsevier: 2010; pp. [8] 64 and 94 figures. Brain. (2010) 133:2838–44. doi: 10.1093/brain/awq270 20928945

[57] De Carvalho M Barkhaus PE Nandedkar SD Swash M. Motor unit number estimation (MUNE): where are we now? Clin Neurophysiol. (2018) 129:1507–16. doi: 10.1016/j.clinph.2018.04.748, PMID: 29804042

[58] Rothwell JC Hallett M Berardelli A Eisen A Rossini P Paulus W. Magnetic stimulation: motor evoked potentials. The International Federation of Clinical Neurophysiology. Electroencephalogr Clin Neurophysiol Suppl. (1999) 52:97–103. 10590980

[59] Ryan JJ Lopez SJ. Wechsler adult intelligence scale-III In: Dorfman WI Hersen M, editors. Understanding psychological assessment. Boston, MA: Springer US (2001). 19–42.

[60] Bergner M Bobbitt RA Carter WB Gilson BS. The sickness impact profile: development and final revision of a health status measure. Med Care. (1981) 19:787–805. doi: 10.1097/00005650-198108000-00001, PMID: 7278416

[61] Ashworth B. Preliminary trial of carisoprodol in multiple sclerosis. Practitioner. (1964) 192:540–2. 14143329

[62] Melzack R. The McGill pain questionnaire: major properties and scoring methods. Pain. (1975) 1:277–99. doi: 10.1016/0304-3959(75)90044-5, PMID: 1235985

[63] R Core Team. R: a language and environment for statistical computing. (2023). Available online at: https://www.r-project.org/ (accessed April 25, 2025)

[64] Apolloni S Amadio S Fabbrizio P Morello G Spampinato AG Latagliata EC . Histaminergic transmission slows progression of amyotrophic lateral sclerosis. J Cachexia Sarcopenia Muscle. (2019) 10:872–93. doi: 10.1002/jcsm.12422, PMID: 31020811 PMC6711424

[65] Latif S Kang YS. Change in cationic amino acid transport system and effect of lysine pretreatment on inflammatory state in amyotrophic lateral Sclerosis cell model. Biomol Ther. (2021) 29:498–505. doi: 10.4062/biomolther.2021.037, PMID: 33935047 PMC8411026

[66] Nabavi SM Arab L Jarooghi N Bolurieh T Abbasi F Mardpour S . Safety, feasibility of intravenous and intrathecal injection of autologous bone marrow–derived mesenchymal stromal cells in patients with amyotrophic lateral sclerosis: an open-label phase I clinical trial. Cell J. (2019) 20:592–8. doi: 10.22074/cellj.2019.5370, PMID: 30124008 PMC6099146

[67] Nabavi SM Karimi SH Arab L Sanjari L Mardpour S Azimian V . Safety and efficacy of allogeneic adipose tissue mesenchymal stromal cells in amyotrophic lateral sclerosis patients: single-center, prospective, open-label, single-arm clinical trial with long-term follow-up. Cell J. (2021) 23:772–8. doi: 10.22074/cellj.2021.7984, PMID: 34979067 PMC8753106

[68] Berry JD Cudkowicz ME Windebank AJ Staff NP Owegi M Nicholson K . NurOwn, phase 2, randomized, clinical trial in patients with ALS: safety, clinical, and biomarker results. Neurology. (2019) 93:e2294–305. doi: 10.1212/WNL.0000000000008620, PMID: 31740545 PMC6937497

[69] Cudkowicz ME Lindborg SR Goyal NA Miller RG Burford MJ Berry JD . A randomized placebo-controlled phase 3 study of mesenchymal stem cells induced to secrete high levels of neurotrophic factors in amyotrophic lateral sclerosis. Muscle Nerve. (2022) 65:291–302. doi: 10.1002/mus.27472, PMID: 34890069 PMC9305113

[70] Siwek T Jezierska-Woźniak K Maksymowicz S Barczewska M Sowa M Badowska W . Repeat administration of bone marrow-derived mesenchymal stem cells for treatment of amyotrophic lateral sclerosis. Med Sci Monit. (2020) 26:e927484. doi: 10.12659/MSM.927484, PMID: 33301428 PMC7737405

[71] Barczewska M Maksymowicz S Zdolińska-Malinowska I Siwek T Grudniak M. Umbilical cord mesenchymal stem cells in amyotrophic lateral Sclerosis: an original study. Stem Cell Rev Rep. (2020) 16:922–32. doi: 10.1007/s12015-020-10016-7, PMID: 32725316 PMC7456414

[72] Syková E Rychmach P Drahorádová I Konrádová Š Růžičková K Voříšek I . Transplantation of mesenchymal stromal cells in patients with amyotrophic lateral sclerosis: results of phase I/IIa clinical trial. Cell Transplant. (2017) 26:647–58. doi: 10.3727/096368916X693716, PMID: 27938483 PMC5661219

