# Supplementary material for: Adipose-derived mesenchymal stem cells for the treatment of Amyotrophic Lateral Sclerosis. A phase I/II safety and efficacy clinical trial
Source: Front Neurol. 2025 Oct 8;16:1655124. doi: 10.3389/fneur.2025.1655124 (PMC12540107; doi:10.3389/fneur.2025.1655124)

Adipose-derived mesenchymal stem cells for the treatment of Amyotrophic Lateral Sclerosis. A phase I/II safety and efficacy clinical trial

Supplementary material

Figure S1. Baseline ALSFRS-r scores, by initial randomization group

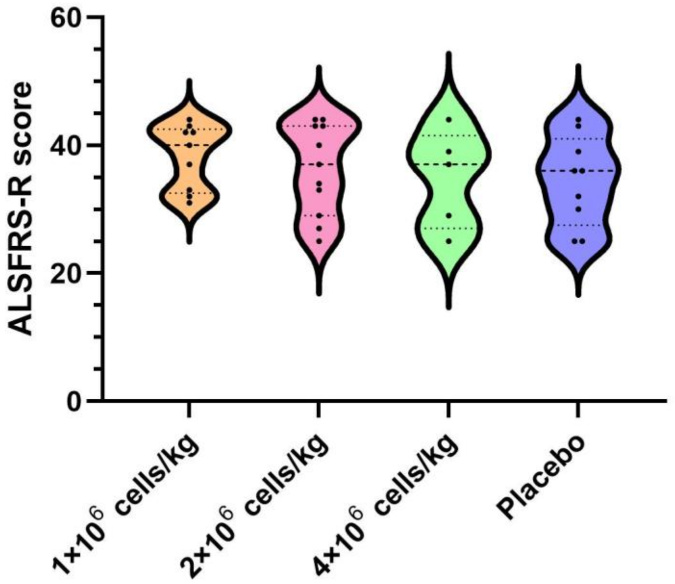

Figure S2. Changes in total ALSFRS-R scores after treatment administration (AdMSC or placebo). Mean and IC 95% of the results based on the initial randomization group are presented.

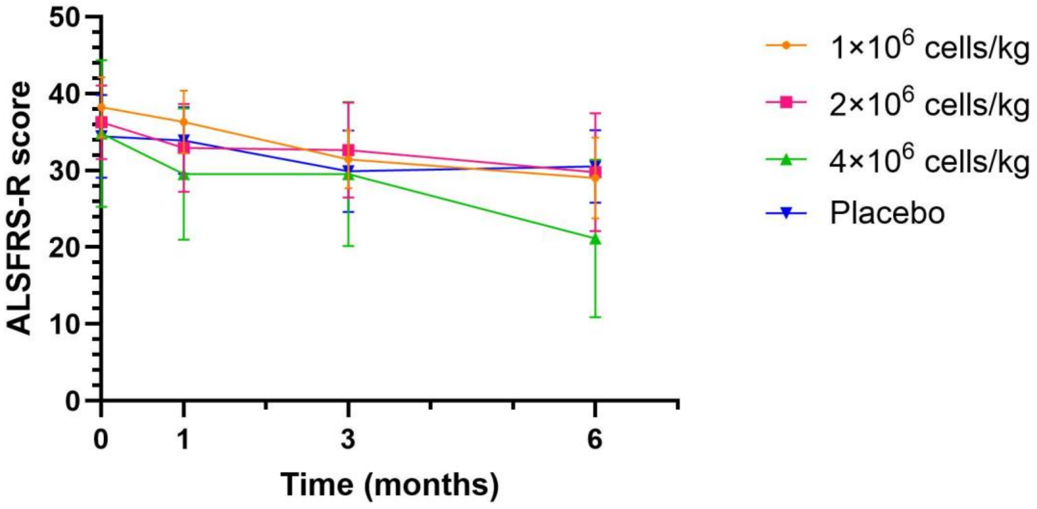

Figure S3. Changes in total ALSFRS-R scores after treatment administration (AdMSC or placebo). Individual trajectories of each patient are represented with thin lines, and the LOESS regression for each treatment group is represented with thick lines.

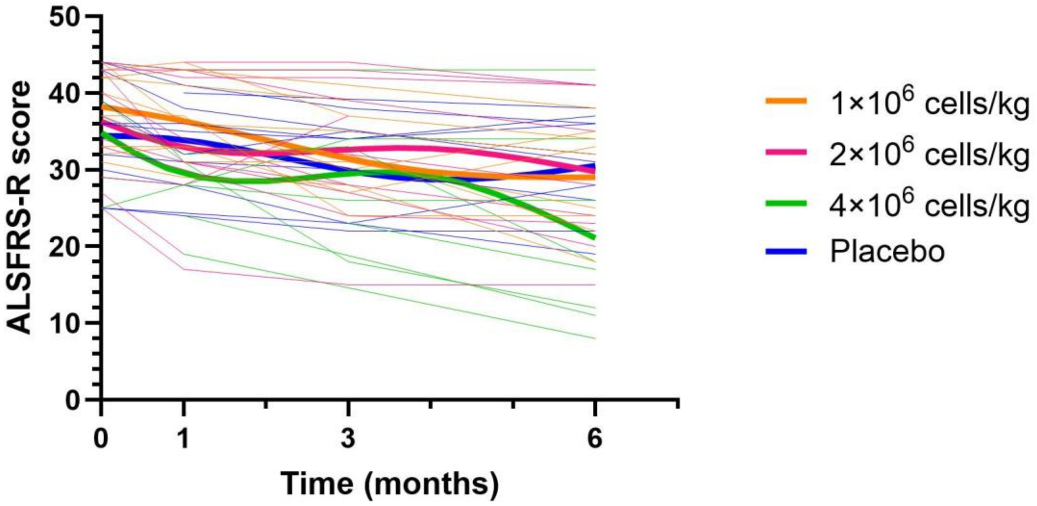

Figure S4. Changes in total ALSFRS-R scores in the patients initially randomized to placebo, after their relocation and treatment with three different doses of AdMSC. Mean and IC 95% of the results are presented.

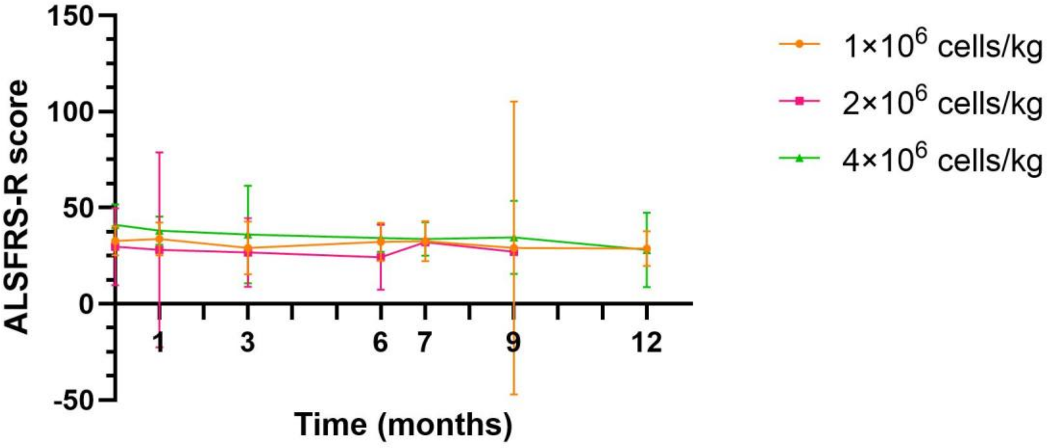

Figure S5. Changes in the FVC after treatment administration (AdMSC or placebo). Mean and IC 95% of the results based on the initial randomization group are presented.

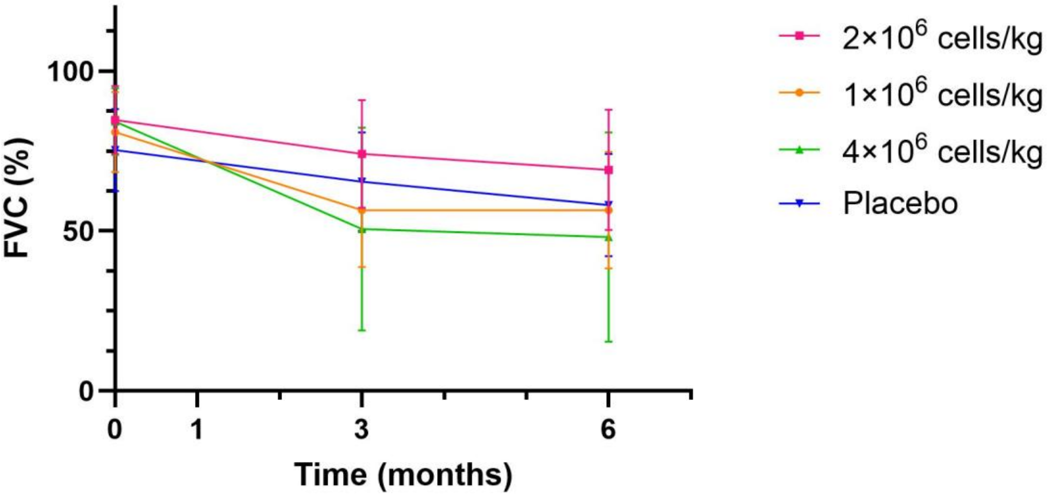

Figure S6. Changes in the neurophysiological index after treatment administration (AdMSC or placebo) (A. Left Member, B. Right member). Mean and IC 95% of the results based on the initial randomization group are presented.

A

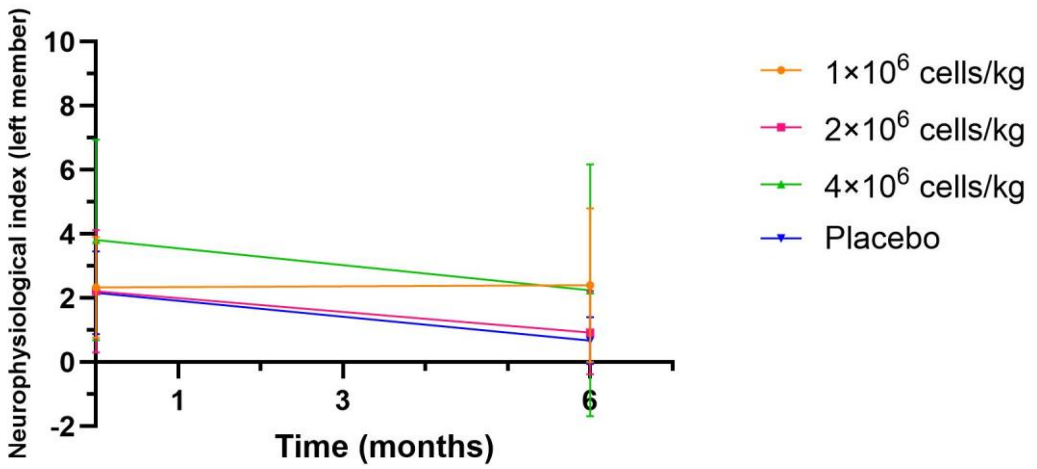

B

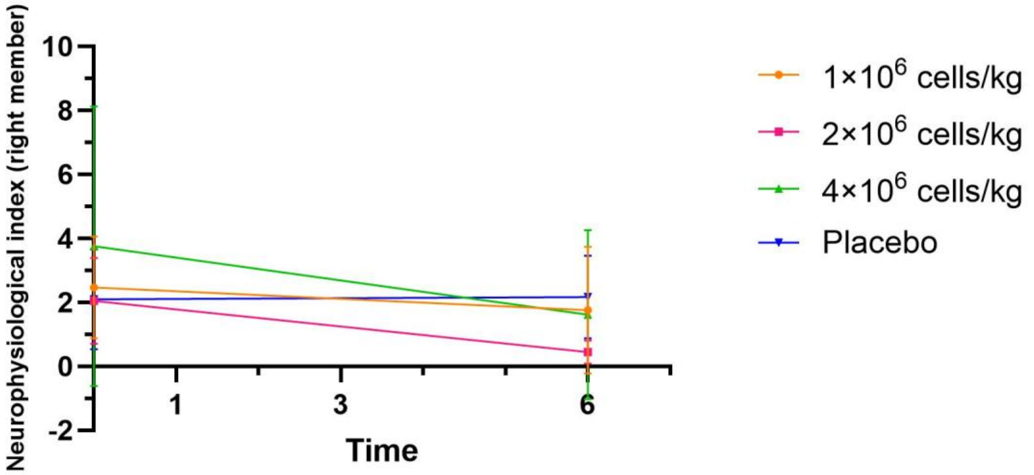

Supplement: Supplementary file 1 [file Data_Sheet_1.PDF]
